# Proteomic Analysis of Pediatric Hemophagocytic Lymphohistiocytosis: a Comparative Study with Healthy Controls, Sepsis, Critical Ill, and Active Epstein-Barr virus Infection to Identify Altered Pathways and Candidate Biomarkers

**DOI:** 10.1007/s10875-023-01573-w

**Published:** 2023-08-31

**Authors:** Xun Li, Ting Luo, Haipeng Yan, Longlong Xie, Yufan Yang, Ling Gong, Zhexuan Tang, Minghui Tang, Xinping Zhang, Jiaotian Huang, Mincui Zheng, Zhenya Yao, Ping Zang, Desheng Zhu, Zhenghui Xiao, Xiulan Lu

**Affiliations:** 1 Pediatrics Research Institute of Hunan Province & Department of Pediatric Intensive Care Unit & Hunan Provincial Key Laboratory of Emergency Medicine for Children, Hunan Children’s Hospital, Changsha, China; 2 General Emergency Ward & Hunan Provincial Key Laboratory of Emergency Medicine for Children, Hunan Children’s Hospital, Changsha, China; 3 Department of Pediatric Intensive Care Unit & Hunan Provincial Key Laboratory of Emergency Medicine for Children, Hunan Children’s Hospital, Changsha, China; 4 Department of Pediatric Hematology, Hunan Children’s Hospital, Changsha, China

**Keywords:** Hemophagocytic lymphohistiocytosis, proteomic, children, sepsis, Epstein-Barr virus, biomarker

## Abstract

Hemophagocytic lymphohistiocytosis (HLH) is a life-threatening hyperinflammatory syndrome characterized by excessive activation of the immune system, along with uncontrolled proliferation of activated macrophages and lymphocytes. The clinical features of HLH often overlap with the clinical features of other severe inflammatory conditions such as sepsis, hindering accurate and timely diagnosis. In this study, we performed a data-independent acquisition mass spectrometry-based plasma proteomic analysis of 33 pediatric patients with HLH compared with four control groups: 39 healthy children, 43 children with sepsis, 39 children hospitalized in the pediatric intensive care unit without confirmed infections, and 21 children with acute Epstein-Barr virus infection. Proteomic comparisons between the HLH group and each of the control groups showed that HLH was characterized by alterations in complement and coagulation cascades, neutrophil extracellular trap formation, and platelet activation pathways. We identified eight differentially expressed proteins in patients with HLH, including plastin-2 (LCP1), vascular cell adhesion protein 1, fibrinogen beta chain, fibrinogen gamma chain, serum amyloid A-4 protein, extracellular matrix protein 1, apolipoprotein A-I, and albumin. LCP1 emerged as a candidate diagnostic marker for HLH with an area under the curve (AUC) of 0.97 in the original cohort and an AUC of 0.90 (sensitivity = 0.83 and specificity = 1.0) in the validation cohort. Complement C1q subcomponent subunit B was associated with disease severity in patients with HLH. Based on comparisons with multiple control groups, this study provides a proteomic profile and candidate biomarkers of HLH, offering researchers novel information to improve the understanding of this condition.

**Supplementary Information:**

The online version contains supplementary material available at 10.1007/s10875-023-01573-w.

## Introduction

Hemophagocytic lymphohistiocytosis (HLH) is a life-threatening hyperinflammatory syndrome characterized by excessive activation of the immune system, along with uncontrolled proliferation of activated macrophages and lymphocytes [1, 2]. Activated lymphocytes and macrophages generate a cytokine storm that induces hemophagocytosis and tissue phagocytosis, ultimately resulting in multiple organ dysfunction syndrome (MODS) and possible death. Early diagnosis and treatment of HLH are necessary to improve patient outcomes; however, the clinical features of HLH often overlap with the clinical features of other severe inflammatory conditions, such as sepsis, hindering accurate and timely diagnosis. There is an urgent need to identify biomarkers and molecular signatures that can distinguish HLH from other conditions and guide appropriate therapeutic interventions.

Prior investigations of HLH have explored potential biomarkers and expression levels of inflammatory proteins [3–5]. However, most of these studies selected proteins based on previous research findings, clinical relevance, or mechanistic hypotheses. A comprehensive protein profile for HLH has not been elucidated. By examining the global protein expression patterns in patient samples, proteomic analyses can offer insights into the molecular pathways and biological processes associated with HLH, leading to a better understanding of its pathogenesis and progression, along with opportunities to identify diagnostic and prognostic biomarkers. Therefore, the present study explored the plasma proteomic profiles of patients with HLH and compared them with the profiles of healthy controls and patients with other inflammatory or critical conditions.

The selection of disease control groups in this study was based on clinical situations in which HLH must be distinguished from other conditions. First, the clinical features of sepsis and HLH can sometimes be difficult to distinguish, and these conditions may share triggering factors [6, 7]; nevertheless, their pathophysiological mechanisms and treatment plans are distinct. To reveal protein-level differences between sepsis and HLH, the first disease control group comprised patients with sepsis. Second, many cases of HLH are diagnosed in the intensive care unit [8]. Because diagnosis and management of HLH are challenging, HLH is presumably an undetected cause of death in some critically ill patients [9]. We speculated that patients in the pediatric intensive care unit (PICU) with infections may overlap with patients in the sepsis group; therefore, the second disease control group comprised patients from the PICU without confirmed infections. Finally, because Epstein-Barr virus (EBV) is a known trigger and one of the main causes of secondary HLH in Asian regions [10], therefore, third disease control group comprised patients who had EBV infection without HLH. By identifying differentially expressed proteins (DEPs) and analyzing their involvement in various biological pathways, we sought to discover potential biomarkers for HLH and offer a comprehensive proteomic profile of HLH for researchers pursuing a better understanding of this condition.

## Methods

### Study Design and Population

This case-control study involved patients from Hunan Children’s Hospital. Patient enrollment was performed from May 2018 to November 2021. The proteomic analysis was conducted on December 2021, and the validation study was conducted on July 2023. Pediatric patients diagnosed with HLH during the enrollment period were assigned to the HLH group. Four control groups were established: one healthy control group and three disease control groups (sepsis group, PICU non-infection group, and EBV non-HLH group). The healthy control group comprised children who visited the hospital for health examinations and had no detected diseases. The sepsis group comprised patients who were diagnosed with sepsis and hospitalized in the PICU. The PICU non-infection group comprised patients without confirmed infection or sepsis who were hospitalized in the PICU. The EBV non-HLH group comprised patients who were diagnosed with acute EBV infection and did not have HLH. Patients in whom HLH could not be definitively ruled out were excluded from the control groups. Patients in the HLH and control groups were matched according to age and sex. Patients in the HLH group were classified into three subgroups based on the potential trigger of HLH: HLH triggered by EBV (EBV-HLH), malignancy-associated HLH (HLH-malignancy), and HLH triggered by other infections (HLH-other infection). HLH was diagnosed using HLH-2004 criteria [1], and sepsis was diagnosed using the 2012 Surviving Sepsis Campaign criteria [11].

### Sample and Data Collection

Blood samples were collected in ethylenediaminetetraacetic acid anticoagulant tubes either during the first blood draw for the admission blood tests (PICU non-infection group) or within 24 h after the diagnosis of HLH, sepsis, or EBV infection. After blood collection, the plasma was immediately separated and stored at −80°C. Demographic and clinical data were extracted from electronic medical records. A chart review was performed to confirm group allocation.

### Data-independent Acquisition-based Proteomic Analysis

Data-independent acquisition (DIA)-based quantitative proteomic analysis (Shanghai Biotree Co., Ltd, Shanghai, China) was performed for all five groups of plasma samples. Plasma samples were prepared for mass spectrometry (MS) as previously described [12]. Details of the DIA-based proteomic analysis are presented in Supplementary information 1.

### Targeted MS-based Validation

Candidate protein markers were validated in an independent cohort using the parallel reaction monitoring (PRM)-based targeted MS approach (Shanghai Biotree Co., Ltd.) [13]. The validation cohort included an HLH group of 6 children with secondary HLH (3 with EBV-HLH, 2 with HLH-other infection, and 1 with HLH-malignancy) and a non-HLH group of 16 children without HLH. The non-HLH group comprised six patients with sepsis, four patients with EBV infection, two patients with leukemia, and four healthy children with no known diseases. The HLH and non-HLH groups were matched according to age and sex. The plasma levels of candidate protein markers were compared between the HLH and non-HLH groups. Comparisons between the HLH group and subgroups within the non-HLH group were not conducted because these subgroups had small sample sizes that would have led to limited statistical power.

### General Statistical Methods

The *t*-test, chi-squared test, and Wilcoxon rank-sum test were used for between-group comparisons. Multiple comparisons of demographic and clinical characteristics were adjusted using the Bonferroni method. All tests were two-tailed, and the type 1 error rate was set at 5%. Missing data were not imputed. Statistical analyses were performed using SAS ver. 9.4 (SAS Institute, Cary, NC, USA) and R ver. 4.1.3 (R Core Team, Vienna, Austria). Figures were generated using R ver. 4.1.3 and GraphPad Prism ver. 8 (GraphPad Software, Inc., La Jolla, CA, USA).

### Differential Expression Analysis and Heatmap Presentation

The *t*-test was used to examine proteins that were differentially expressed between comparison groups. To control the false discovery rate, multiple testing was adjusted using the Benjamini–Hochberg (BH) method. DEPs were defined as proteins that had different expression levels between the HLH group and control groups with a BH-adjusted *P* value of <0.05 and fold change of >1.5 or <0.67. The “pheatmap” function in R was used to display DEPs.

### Pathway Enrichment Analysis and Network Visualization

To obtain an overview of the enriched pathways in HLH compared with non-HLH, proteomic data from the four control groups were pooled and compared with proteomic data from the HLH group. Because the purpose of this step was to determine the overall change in proteomic profiles among patients with HLH rather than identify potential biomarkers, a more lenient threshold comprising a *P* value of <0.05, BH-adjusted *P* value of <0.1, and fold change of >1.3 or <0.77 was chosen for heatmap presentation and enrichment analysis. The network of enriched functions and pathways was visualized using Cytoscape ver. 3.8.2 (Supplementary information 1).

### Receiver Operating Characteristic Analyses of Potential Biomarkers

Receiver operating characteristic (ROC) analyses for potential diagnostic and prognostic markers of HLH were performed using GraphPad Prism ver. 8, and the area under the curve (AUC) was calculated. The sensitivity and specificity corresponding to the maximum Youden’s index are presented.

### Correlations Between DEPs and Clinical Laboratory Test Results

To investigate the clinical relevance of the most salient DEPs, the correlations between DEPs and diagnostic parameters for HLH were examined using Spearman’s correlation test, and correlation coefficients (*r*_*s*_) were calculated.

## Results

### Study Population

**Fig. 1:**
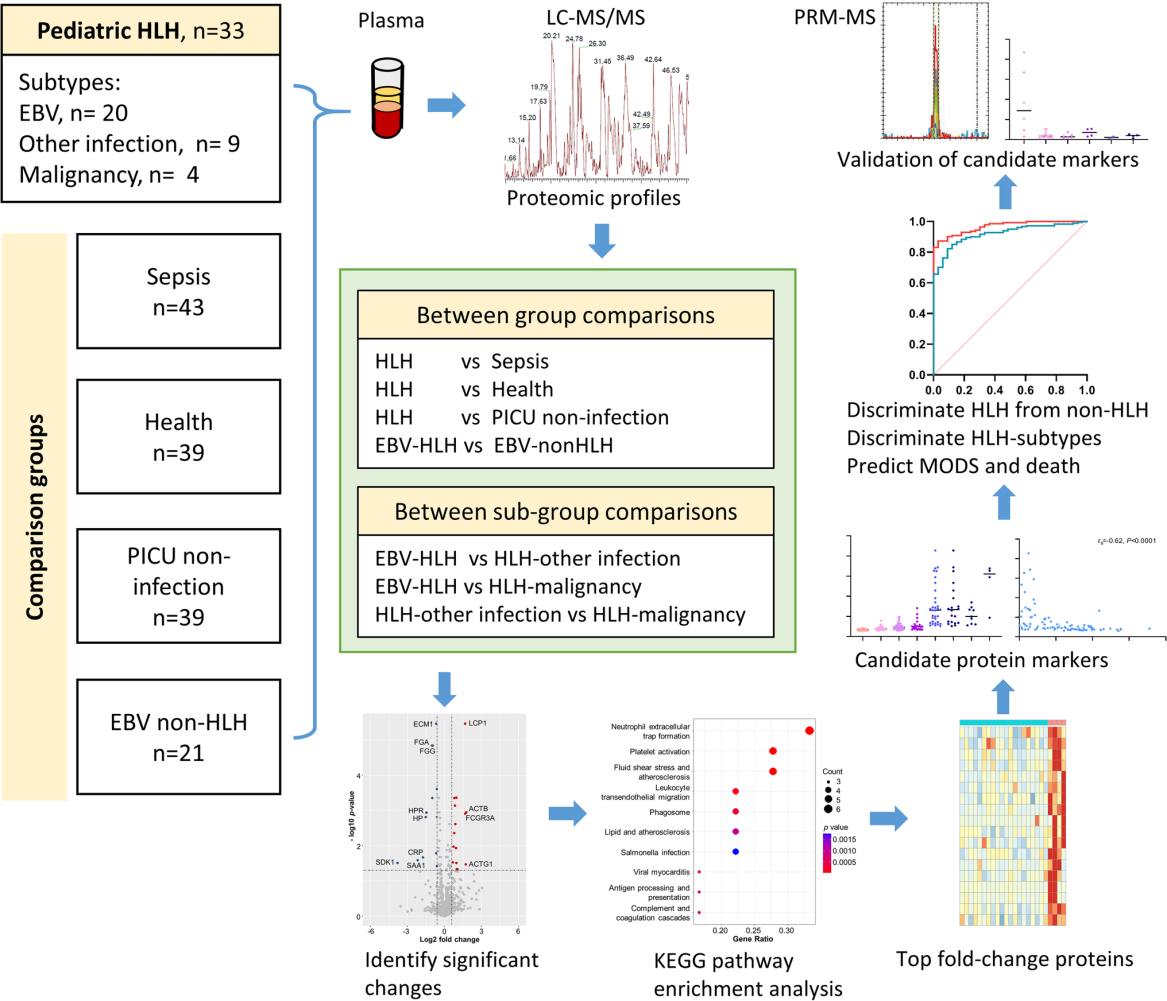
**Study flow chart**

This study included 33 pediatric patients with HLH (20 with EBV-HLH, 4 with HLH-malignancy, and 9 with HLH-other infection). The four comparison groups were the sepsis group (*n* = 43), healthy control group (*n* = 39), PICU non-infection group (*n* = 39), and EBV non-HLH group (*n* = 21) (Fig. 1). Table 1 shows the demographic and clinical characteristics of the HLH group and the disease control groups. Between-group comparisons showed that the age and sex distributions were not significantly different between the HLH group and each of the control groups (*P* > 0.0167) (Table 1). Among the four disease groups, the HLH group showed the highest rate of non-recovery or death at hospital discharge. During hospital admission, the HLH group had lower neutrophil counts and white blood cell counts compared with the disease control groups (*P* < 0.0167). Supplementary Table S1 shows the demographic and clinical characteristics of the HLH subgroups. There were no significant differences in age and sex distribution among the HLH subgroups (*P* > 0.0167) (Supplementary Table S1).

**Table 1:** Demographic and clinical characteristics of the HLH group and disease control groups

Variable	HLH (*n* = 33)	Sepsis (*n* = 43)	PICU non-infection (*n* = 39)	EBV (*n* = 21)	*P*_HLH vs_		
					Sepsis	PICU non-infection	EBV
Age, year	2 (0, 3)	2 (0, 5)	2 (1, 3)	3 (1, 4)	0.5185	0.7250	0.0516
Sex							
Female	19 (57.6)	18 (41.9)	23 (59.0)	11 (52.4)	0.1743	0.9045	0.7080
Male	14 (42.4)	25 (58.1)	16 (41.0)	10 (47.6)			
Clinical outcome at hospital discharge							
Recovery/improvement	19 (57.6)	35 (81.4)	36 (92.3)	20 (95.2)	0.0232	**0.0005**	**0.0026**
Non-recovery/death	14 (42.4)	8 (18.6)	3 ( 7.7)	1 ( 4.8)			
Complications during hospitalization							
DIC							
No	26 (78.8)	35 (81.4)	39 ( 100)	20 (95.2)	0.7771	**0.0029**	0.1310
Yes	7 (21.2)	8 (18.6)	0	1 ( 4.8)			
Shock							
No	27 (81.8)	24 (55.8)	37 (94.9)	20 (95.2)	0.0168	0.1312	0.2269
Yes	6 (18.2)	19 (44.2)	2 ( 5.1)	1 ( 4.8)			
Heart failure							
No	29 (87.9)	39 (90.7)	37 (94.9)	20 (95.2)	0.7215	0.4029	0.6377
Yes	4 (12.1)	4 ( 9.3)	2 ( 5.1)	1 ( 4.8)			
AKI							
No	30 (90.9)	39 (90.7)	38 (97.4)	20 (95.2)	1.0000	0.3266	1.0000
Yes	3 ( 9.1)	4 ( 9.3)	1 ( 2.6)	1 ( 4.8)			
Respiratory failure							
No	17 (51.5)	24 (55.8)	36 (92.3)	18 (85.7)	0.7094	**<0.0001**	**0.0103**
Yes	16 (48.5)	19 (44.2)	3 ( 7.7)	3 (14.3)			
ARDS							
No	30 (90.9)	39 (90.7)	39 ( 100)	21 ( 100)	1.0000	0.0915	0.2736
Yes	3 ( 9.1)	4 ( 9.3)	0	0			
MODS							
No	14 (42.4)	18 (41.9)	33 (84.6)	19 (90.5)	0.9606	**0.0002**	**0.0004**
Yes	19 (57.6)	25 (58.1)	6 (15.4)	2 ( 9.5)			
Laboratory tests during hospital admission							
Hemoglobin, g/L	88.0 (75.0, 104.0)	91.0 (77.0, 108.0)	110.0 (85.0, 117.0)	105.0 (95.0, 121.0)	0.7098	0.0827	0.0262
Neutrophil count, ×10^9^/L	1.11 (0.83, 2.29)	6.62 (2.33, 13.71)	6.98 (3.07, 9.41)	3.53 (1.73, 5.03)	**<0.0001**	**<0.0001**	**0.0046**
Platelet count, ×10^9^/L	59.0 (34.0, 103.0)	194.0 (100.0, 341.0)	328.0 (244.0, 377.0)	217.0 (52.0, 306.0)	**<0.0001**	**<0.0001**	0.1546
White blood cell count, ×10^9^/L	2.29 (1.60, 4.06)	13.03 (7.76, 17.23)	11.07 (7.75, 12.94)	9.30 (5.20, 15.00)	**<0.0001**	**<0.0001**	**0.0006**
Lymphocyte count, ×10^9^/L	1.06 (0.54, 2.20)	1.91 (1.37, 6.90)	2.87 (2.09, 3.93)	4.84 (1.09, 6.67)	**0.0030**	**0.0002**	0.0202
C-reactive protein, mg/L	14.30 (4.28, 34.99)	87.76 (26.34, 129.26)	<0.50 (<0.50, 3.68)	3.48 (1.80, 22.78)	**0.0003**	**<0.0001**	0.2812

*AKI* acute kidney injury, *ARDS* acute respiratory distress syndrome, *DIC* disseminated intravascular coagulation, *EBV* Epstein-Barr virus, *MODS* multiple organ dysfunction syndrome, *HLH* hemophagocytic lymphohistiocytosis, *PICU* pediatric intensive care unit

Values were presented as *n* (%) or median (quartile 1, quartile 3). Bold values were statistically significant (*P* < 0.0167) after Bonferroni correction for multiple comparisons (α’ = 0.05/3 = 0.0167).

### Quality Control Results of Proteomic Analysis

In total, 1321 proteins were identified and quantified in the 5 groups. Quality control analysis of the indexed retention time/retention time, data points per peak, and total ion chromatogram showed consistent stability of the MS platform (Supplementary Fig. S1). Pearson’s correlation coefficients were calculated to validate the correlations of specific proteins identified by proteomic analysis with the related clinical test results. The validation process involved comparing the quantified levels of proteins identified via proteomic analysis with the corresponding levels obtained from clinical tests conducted on similar sampling dates. For example, the Pearson correlation coefficient was 0.78 (*P* <0.0001) for the relationship between the proteomic-quantified fibrinogen alpha chain and the clinically measured fibrinogen level (Supplementary Fig. S2), demonstrating that the proteomic analysis platform was reliable.

### Proteomic Characteristics of HLH and Network of Enriched Functions

**Fig. 2:**
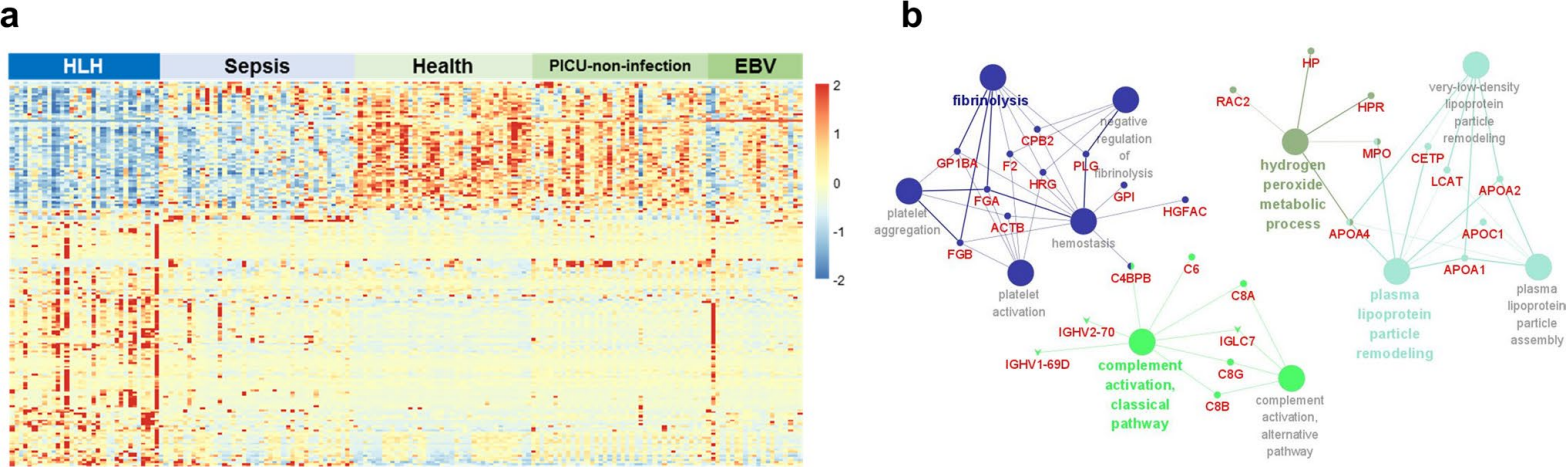
**Proteomic characteristics of HLH and network of enriched functions.** (**a**) Heatmap of proteins with a *P* value of <0.05, BH-adjusted *P* value of <0.1, and fold change of >1.3 or <0.77 between HLH and non-HLH patients. (**b**) Network of enriched functions in HLH versus non-HLH patients

In the comparison of proteomic profiles between HLH and non-HLH, 194 proteins displayed *P valu*es of <0.05, adjusted *P* values of <0.1, and fold change of >1.3 or <0.77 (Fig. 2a, Supplementary Table S2). Changes in plasma proteins among patients with HLH were mainly enriched in four functional clusters: fibrinolysis, complement activation, hydrogen peroxide metabolic processes, and plasma lipoprotein particle remodeling (Fig. 2b).

### Proteomic Characteristics of HLH Compared with Sepsis

**Fig. 3:**
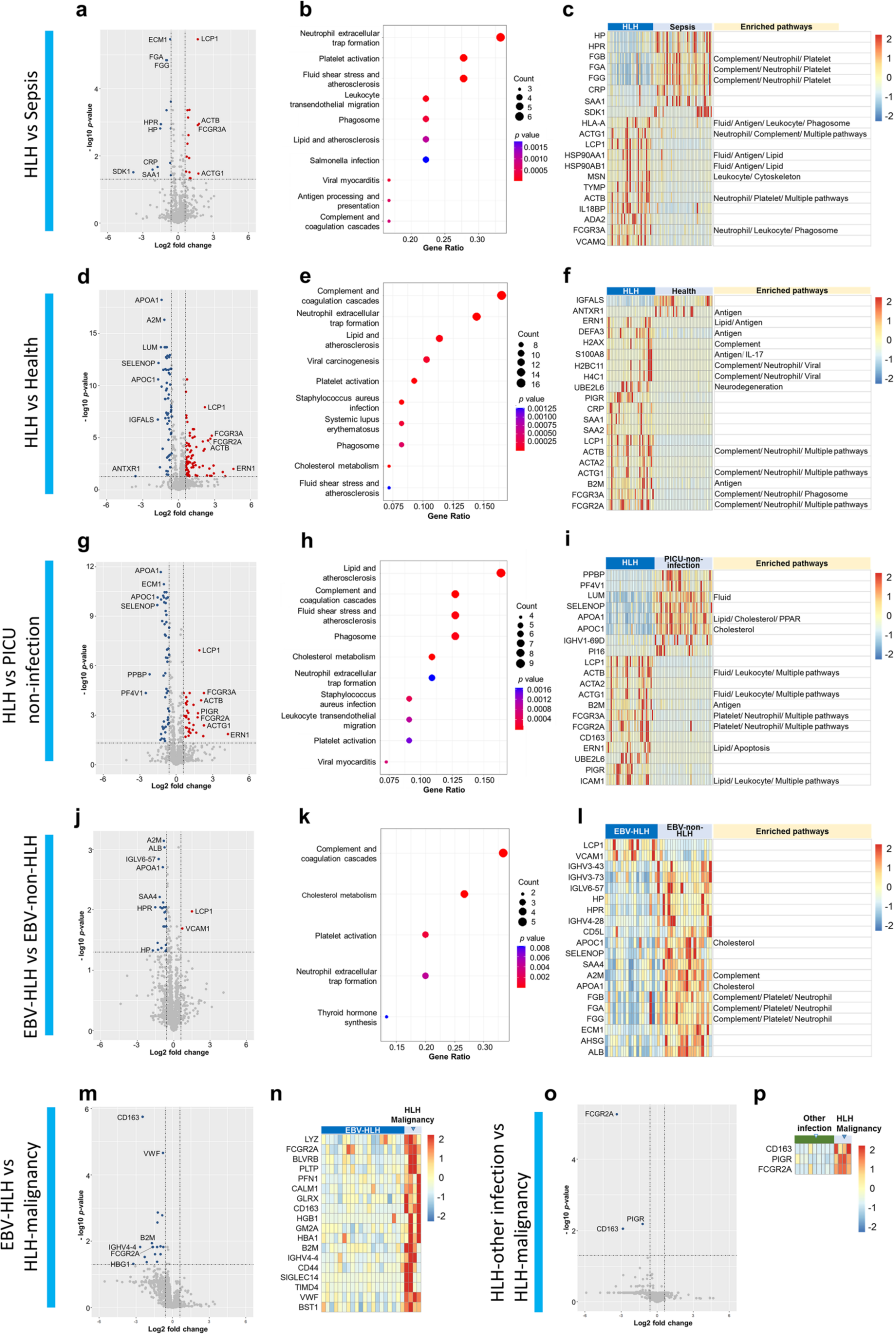
**Proteomic differences between HLH and control groups.** This figure shows the volcano plots, KEGG enriched pathways, and heatmaps of the 20 DEPs with the largest fold changes for comparison between the (**a**–**c**) HLH and sepsis groups, (**d**–**f)** HLH and healthy control groups, (**g**–**i**) HLH and PICU non-infection groups, and (**j**–**l**) EBV-HLH and EBV non-HLH groups. The figure also shows the volcano plots and the heatmaps of the top DEPs for proteomic comparison between the (**m** and **n**) EBV-HLH and HLH-malignancy groups and (**o** and **p**) HLH-other infection and HLH-malignancy groups. The full names corresponding to the abbreviated pathways provided after the heatmap are as follows: Antigen, antigen processing and present; Complement, complement and coagulation cascades; Cholesterol, cholesterol metabolism; Cytoskeleton, regulation of actin cytoskeleton; Fluid, fluid shear stress and atherosclerosis; IL-17, IL-17 signaling pathway; Lipid, lipid and atherosclerosis; Leukocyte, leukocyte transendothelial migration; Neurodegeneration, pathways of neurodegeneration – multiple diseases; Neutrophil, neutrophil extracellular trap formation; Platelet, platelet activation; PPAR, PPAR signaling pathway; Viral, viral carcinogenesis

Comparison of proteomic profiles between sepsis and HLH revealed 28 DEPs, which were mainly involved in pathways related to neutrophil extracellular trap formation, platelet activation, and fluid shear stress and atherosclerosis (Fig. 3a,b, Supplementary Table S3). The 20 DEPs with the largest fold changes were mainly involved in the pathways of neutrophil extracellular trap formation, fluid shear stress and atherosclerosis, and leukocyte transendothelial migration (Fig. 3c). Four proteins showed a >3-fold difference between HLH and sepsis: actin, cytoplasmic 1 (ACTB); actin, cytoplasmic 2 (ACTG1); CD16a antigen (FCGR3A); and plastin-2 (LCP1) (Fig. 3a). Compared with sepsis, the three proteins with the largest decrease in HLH were protein sidekick-1 (SDK1), serum amyloid A-1 protein (SAA1), and C-reactive protein (CRP ) (Fig. 3a).

### Proteomic Characteristics of HLH Compared with Absence of Medical Conditions

To obtain general insights into the differences between children with HLH and healthy children, we compared the proteomic profiles of these two groups and identified 140 DEPs (adjusted *P* < 0.05, fold change >1.5 or <0.67) (Fig. 3d, Supplementary Table S4). Kyoto Encyclopedia of Genes and Genomes (KEGG) enrichment analysis demonstrated that HLH-associated DEPs were mainly involved in pathways related to complement and coagulation cascades, neutrophil extracellular trap formation, lipid and atherosclerosis, viral carcinogenesis, and platelet activation (Fig. 3e). The 20 DEPs with the largest fold changes are shown in Fig. 3f. These DEPs were mainly enriched in the pathways of complement and coagulation cascades/neutrophil extracellular trap formation, antigen processing and presentation, and fluid shear stress and atherosclerosis.

### Proteomic Characteristics of HLH Compared with PICU Admission in the Absence of Infection

Eighty-six DEPs were identified in the comparison between children with HLH and critically ill children without known infections (Fig. 3g, Supplementary Table S5). The 86 DEPs were mainly involved in the pathways of lipid and atherosclerosis, complement and coagulation cascades, fluid shear stress and atherosclerosis, and phagosome (Fig. 3h). The 20 DEPs with the largest fold changes and related pathways are shown in Fig. 3i.

### Proteomic Characteristics of EBV-HLH Compared with EBV Infection in the Absence of HLH

Comparison of the proteomic profiles between EBV-HLH and EBV non-HLH groups identified 27 DEPs (Fig. 3j, Supplementary Table S6), most of which were downregulated in EBV-HLH; the exceptions were LCP1 and vascular cell adhesion protein 1 (VCAM1). The most enriched pathways were related to complement and coagulation cascades and cholesterol metabolism (Fig. 3k). The three DEPs with the largest differences were haptoglobin (HP), haptoglobin-related protein (HPR), and LCP1 (Fig. 3j,l).

### Characterization of HLH Subtypes

Eighteen DEPs were identified in the comparison between the EBV-HLH and HLH-malignancy groups, and all were expressed at lower levels in the EBV-HLH group (Fig. 3m,n). The three DEPs with the largest differences were hemoglobin subunit gamma-1 (HBG1), immunoglobulin heavy variable 4-4 (IGHV4-4), and scavenger receptor cysteine-rich type 1 protein M130 (CD163) (Fig. 3m,n). No significantly enriched pathways were detected in the KEGG enrichment analysis, possibly because of the small sample size and small number of identified DEPs.

Compared with the HLH-malignancy group, three DEPs were significantly decreased in the HLH-other infection group (Fig. 3o), including CD32 (FCGR2A), CD163, and polymeric immunoglobulin receptor (PIGR) (Fig. 3o,p). Compared with the EBV-HLH and HLH-other infection group, FCGR2A and CD163 were significantly increased in the HLH-malignancy group (Fig. 3m,o). No significant DEPs were identified between the HLH-EBV and HLH-other infection groups.

### Significantly Altered Pathways in HLH

**Fig. 4:**
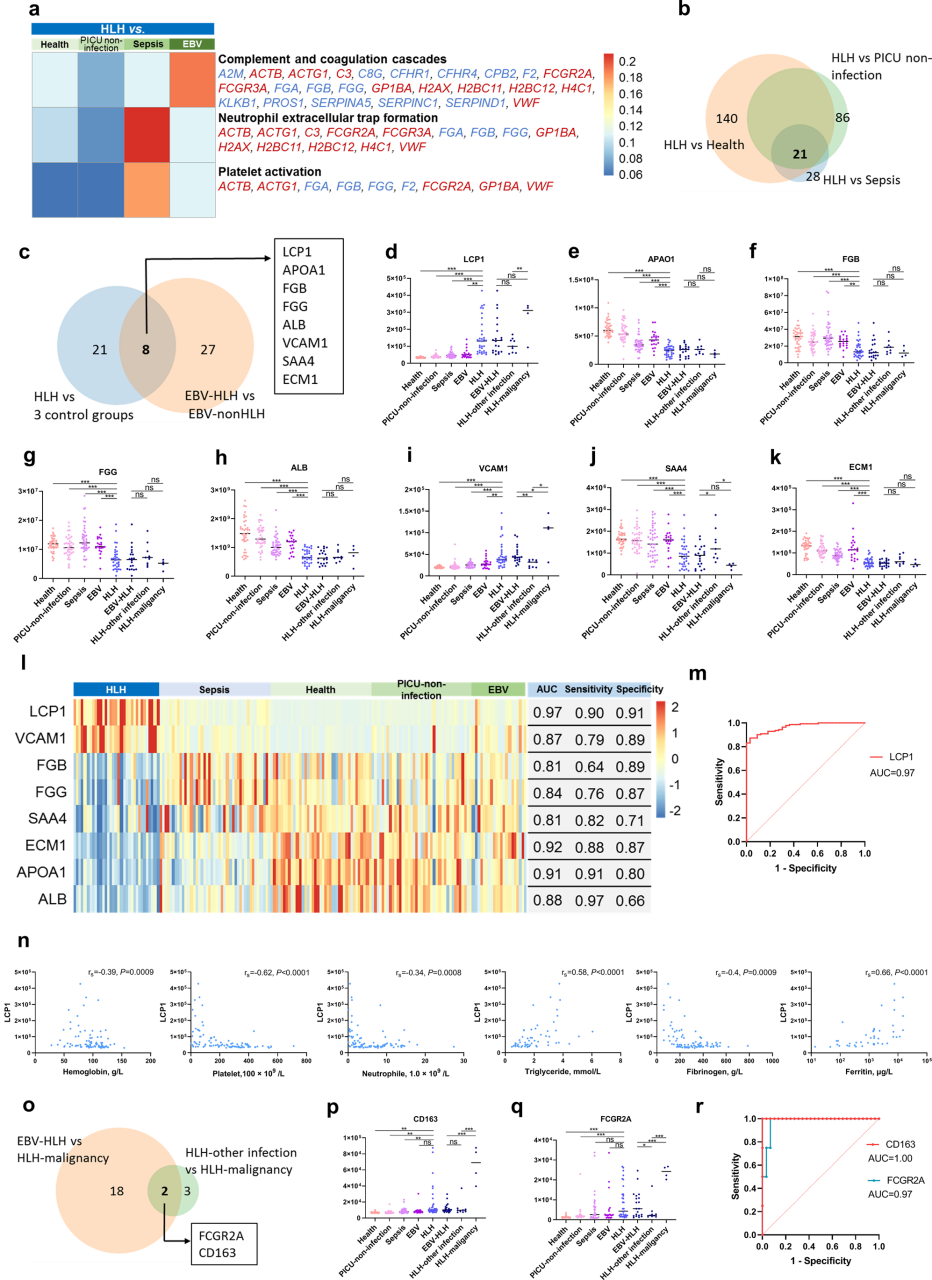
**Proteins and pathways significantly altered in HLH.** (**a**) Pathway enrichment heatmap for pathways significantly altered in HLH compared with four control groups. (**b** and **c**) Venn diagrams showing the numbers of overlapping DEPs between HLH and control groups. Plasma levels of DEPs significantly altered in HLH, including (**d**) LCP1, (**e**) APOA1, (**f**) FGB, (**g**) FGG, (**h**) ALB, (**i**) VCAM1, (**j**) SAA4, and (**k**) ECM1. (**l**) A heatmap with the AUC, sensitivity, and specificity of eight DEPs that were significantly altered in the HLH group compared with non-HLH groups. (**m)** ROC of LCP1 in distinguishing HLH from non-HLH patients. (**n)** Correlations between LCP1 and other clinical diagnostic indicators, including hemoglobin, platelet count, neutrophil count, triglyceride, fibrinogen, and ferritin. (**o**) Venn diagram showing the numbers of overlapping DEPs between subtypes of HLH. Plasma levels of (**p**) CD163 and (**q**) FCGR2A are shown. (**r)** The ROCs of FCGR2A and CD163 in distinguishing malignancy-associate HLH from other types of HLH. ***, *P* < 0.0005; **, *P* < 0.005; * *P,* < 0.05; ns, non-significant, *P* ≥ 0.05

The pathway enrichment heatmap showed that HLH was characterized by alterations in the pathways of complement and coagulation cascades, neutrophil extracellular trap formation, and platelet activation (Fig. 4a). The highest enrichment score for the pathway involved in complement and coagulation cascades was observed in the comparison between the HLH-EBV and EBV non-HLH groups. The highest enrichment scores for the pathways of neutrophil extracellular trap formation and platelet activation were observed in the comparison between the HLH and sepsis groups.

### Significantly Altered Proteins in HLH

Venn diagrams were created to show the number of overlapping DEPs between the HLH and control groups (Fig. 4b,c). Eight mutual DEPs were significantly altered in the HLH group compared with the non-HLH groups, including LCP1, VCAM1, fibrinogen beta chain (FGB), fibrinogen gamma chain (FGG), serum amyloid A-4 protein (SAA4), extracellular matrix protein 1 (ECM1), apolipoprotein A-I (APOA1), and albumin (ALB) (Fig. 4d–l). Among these, LCP1 and VCAM1 were significantly increased in HLH; the other DEPs were significantly decreased in HLH compared with the other non-HLH groups. The AUCs of these DEPs for distinguishing HLH ranged from 0.81 to 0.97 (Fig. 4l). LCP1 showed the highest AUC for distinguishing HLH from non-HLH (AUC = 0.97), with a sensitivity of 0.90 and specificity of 0.91 (Fig. 4l,m). In contrast, the AUCs of hemoglobin, neutrophil count, and platelet count for distinguishing HLH were 0.59 (sensitivity = 0.76, specificity = 0.45), 0.82 (sensitivity = 0.79, specificity = 0.77), and 0.78 (sensitivity = 0.85, specificity = 0.74), respectively.

### LCP1 as a Potential Diagnostic Marker for Pediatric HLH

The most prominent candidate diagnostic marker for pediatric HLH identified in the proteomic analysis was LCP1. The LCP1 level was 3.5-fold higher in HLH than in non-HLH. We also explored the associations between LCP1 and other clinical diagnostic indicators, including hemoglobin, platelet count, neutrophil count, triglycerides, fibrinogen, and ferritin, all of which showed significant correlations (*r*_*s*_ = −0.62 to 0.66; *P* <0.05) (Fig. 4n). The strongest correlation was observed between LCP1 and ferritin (*r*_*s*_ = 0.66, *P* <0.0001) (Fig. 4n).

### FCGR2A and CD163 as Candidate Biomarkers for Malignancy-associated HLH

Because FCGR2A and CD163 were significantly increased in HLH-malignancy (Fig. 4o–q), we explored the AUCs of these two proteins for distinguishing HLH-malignancy and other types of HLH. The AUC were 1.0 (sensitivity = 1 and specificity = 1) for CD163 and 0.97 (sensitivity = 1 and specificity = 0.93 ) for FCGR2A (Fig. 4r).

### Significant Decreased in C1QB in Patients with HLH Who Developed MODS and Early Death

**Fig. 5:**
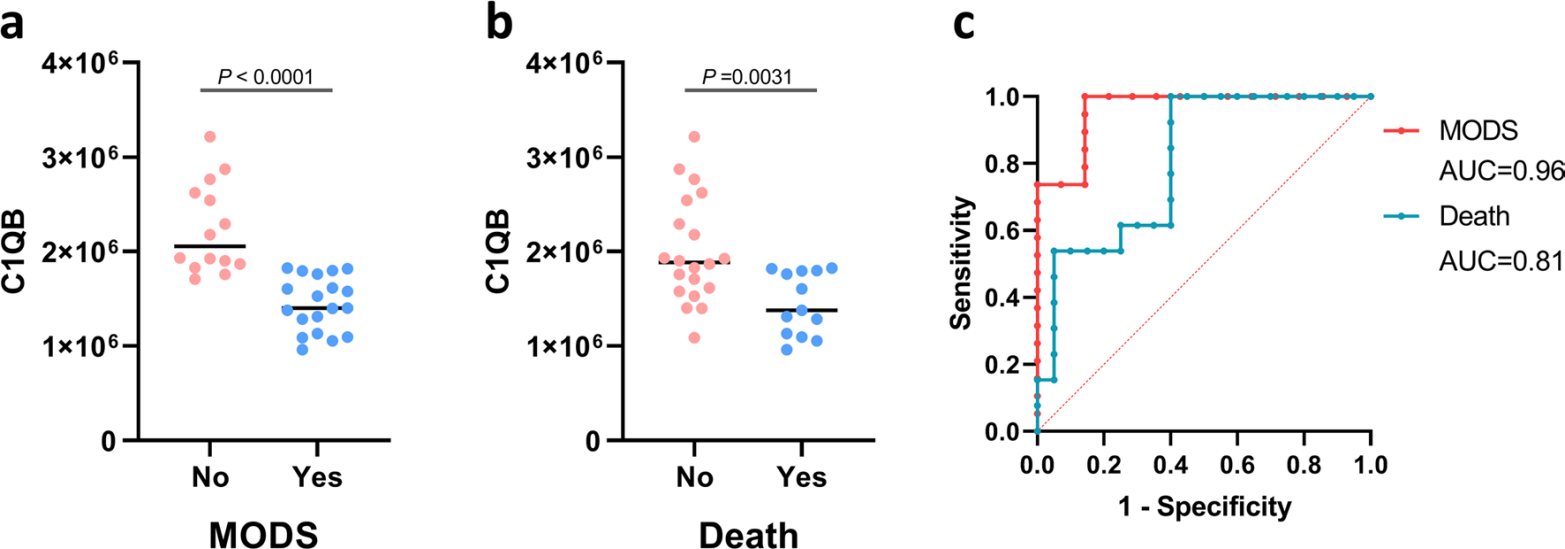
**Association between plasma C1QB level and HLH outcome.** Distribution of C1QB levels according to (**a)** MODS status during hospitalization and (**b**) survival status on day 28. (**c**) The AUCs of C1QB in terms of predicting MODS and early death

We compared proteomic profiles between patients with HLH who did and did not develop MODS, as well as between patients with HLH who survived and died on day 28 after admission. Only one DEP was identified: Complement C1q subcomponent subunit B (C1QB). C1QB was significantly decreased in patients with HLH who developed MODS and early death (Fig. 5a,b), with an AUC of 0.96 (sensitivity = 1, specificity = 0.86) for predicting MODS and an AUC of 0.81 (sensitivity = 0.92, specificity = 0.60) for predicting early death (Fig. 5c).

### Validations of LCP1 and VCAM1

**Fig. 6.**
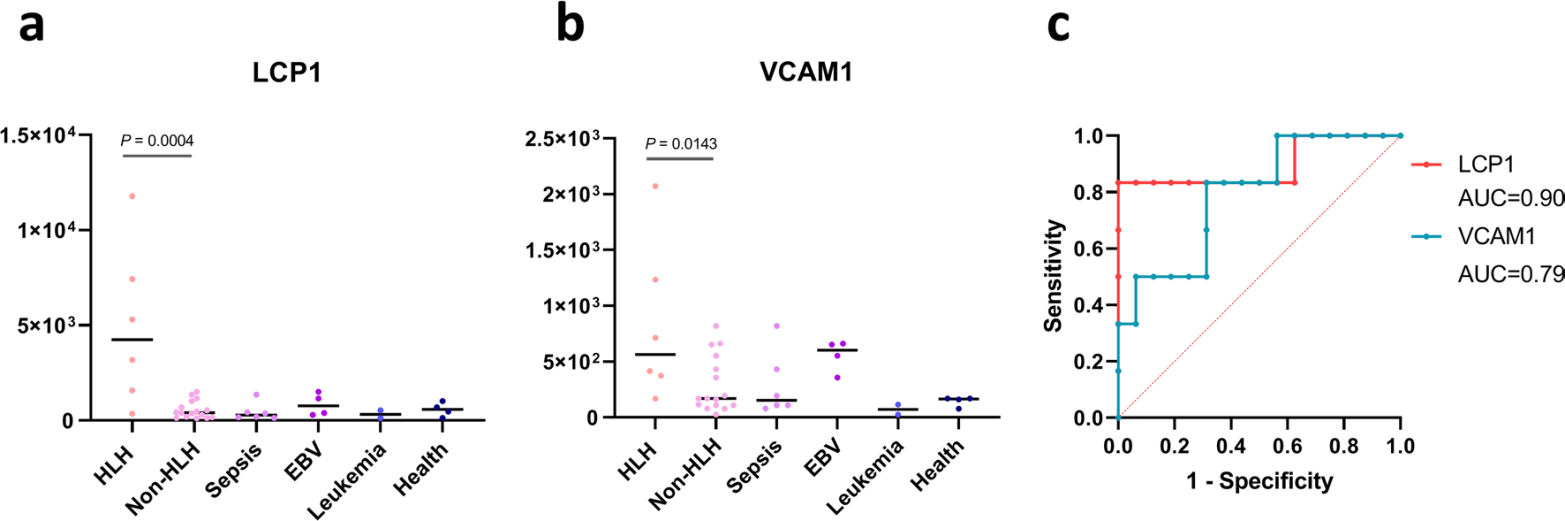
**Validation of LCP1 and VCAM1 by the parallel reaction monitoring-based mass spectrometry approach**. Plasma levels of (**a**) LCP1 and (**b**)VCAM1 among patients with and without HLH from the validation cohort. (**c**) The ROCs of LCP1 and VCAM1 in distinguishing HLH patients in the validation cohort

The diagnostic values of LCP1 and VCAM1 were validated in an independent cohort. The plasma levels of LCP1 and VCAM1 were quantified by the PRM-based MS approach. The HLH group showed significantly higher levels of LCP1 and VCAM1, compared with the non-HLH group (*P* < 0.05, Fig. 6a,b). The AUCs of LCP1 and VCAM1 for distinguishing HLH were 0.90 (sensitivity = 0.83 and specificity = 1.0) and 0.79 (sensitivity = 0.83 and specificity = 0.69), respectively (Fig. 6c).

## Discussion

Proteomic analysis in this study showed that HLH was characterized by alterations in the pathways of complement and coagulation cascades, neutrophil extracellular trap formation, and platelet activation. We identified eight DEPs for HLH in comparison with the healthy group and multiple disease control groups. LCP1 was a candidate diagnostic marker for HLH, FCGR2A and CD163 were potential makers for HLH-malignancy, and C1QB was associated with disease severity (thus helping to predict MODS and early death in patients with HLH).

Proteomic analysis of plasma proteins revealed that HLH was characterized by alterations in proteins associated with the complement and coagulation cascade pathways, neutrophil extracellular trap formation, and platelet activation. Among the DEPs involved in these pathways, FGB and FGG exhibited the most persistent changes compared with all control groups. These findings are consistent with the clinical features of HLH [14]. Decreased neutrophil and platelet counts and hypofibrinogenemia are among the diagnostic criteria for HLH. In patients with HLH, damaged endothelial cells trigger platelet activation and thrombosis in multiple organs, leading to extensive platelet consumption. The release of neutrophil extracellular traps in response to inflammatory signals results in complement activation and platelet activation [15]. Overactivation of these pathways can lead to severe complications associated with HLH. Furthermore, neutrophil targeting has demonstrated benefits in murine models, suggesting a potentially important role for neutrophils in HLH [16].

Among all DEPs identified in this study, LCP1 showed the highest AUC (0.97) for distinguishing HLH, and it performed well in the validation cohort (AUC = 0.90). LCP1 was upregulated in patients with HLH; its expression level was significantly correlated with hemoglobin, platelet count, neutrophil count, triglyceride, fibrinogen, and ferritin, which are diagnostic markers for HLH. LCP1, also known as plastin-2 and L-plastin, is an actin-binding protein that contributes to T-cell activation in response to co-stimulation through the T-cell receptor/CD3 complex and CD2 or CD28 [17]. Additionally, LCP1 modulates the cell surface expression of IL-2 receptor alpha (IL2RA/CD25) and CD69 [17]. Because soluble CD25 (sCD25) serves as a diagnostic marker for HLH, aberrant expression or function of LCP1 might be associated with HLH pathogenesis. Notably, chromosomal aberrations involving LCP1 are implicated in the development of B-cell non-Hodgkin lymphoma [18], which is a potential trigger of HLH [19, 20]. These findings suggest that LCP1 plays a critical role in HLH pathogenesis. Further investigations are needed to evaluate LCP1 as a potential diagnostic marker for HLH and to elucidate the molecular mechanisms associated with LCP1 in HLH.

Comparing the proteomic profiles between HLH and sepsis can provide potential distinguishing markers and may yield insights concerning the pathophysiology of these two diseases. Lin et al. [3] used antibody panels to compare the plasma levels of 135 inflammatory plasma proteins between patients with HLH and patients with severe sepsis or systemic inflammatory response syndrome. They found that the interferon-g (IFN-γ)-regulated chemokines CXCL9, CXCL10, and CXCL11 were significantly altered in the plasma of patients with HLH [3]. Combining these findings with the results of the gene expression study enriched for IFN-γ pathway signatures, the authors concluded that IFN-γ signaling is uniquely elevated in patients with HLH [3]. Similarly, our study showed that IFN-γ signaling pathway proteins (ACTG1, HSP90AB1, and VCAM1) were significantly higher in patients with HLH than in patients with sepsis. Moreover, our study showed that in terms of plasma proteins, the distinctions between HLH and sepsis were mainly enriched in pathways associated with neutrophil extracellular trap formation, platelet activation, and fluid shear stress. Although there is evidence that IFN-γ can promote neutrophil extracellular trap formation [21] and regulate platelet activation [22], the interplay between these pathways may be complex. It would be useful to investigate whether inhibition of the IFN-γ signaling pathway at an early stage can regulate abnormal neutrophil extracellular trap formation and platelet activation in patients with HLH, thereby preventing the development and progression of HLH.

EBV infection is a potential trigger for HLH, particularly in Asian populations. The need for early diagnosis of EBV-HLH has motivated researchers to seek early diagnostic methods [23, 24]. Xie et al. [24] compared the proteomic patterns of plasma exosomal proteins between patients with EBV-HLH and healthy controls, yielding several potential biomarkers for EBV-HLH: CRP, moesin (MSN), galectin-3-binding protein (LGALS3BP), heat shock cognate 71 kDa protein (HSPA8), plasminogen (PLG), and fibronectin 1. Our study showed that although the levels of CRP, MSN, LGALS3BP, HSPA8, and PLG were significantly altered in HLH compared with some control groups, these alterations were not consistently present in all control groups (Supplementary Tables S2–S6). For example, MSN levels were higher in the HLH group than in the sepsis, PICU non-infection, and healthy control groups. However, MSN levels did not significantly differ between the EBV-HLH and EBV non-HLH groups, suggesting that these proteins have a limited ability to detect HLH within a complex pool of non-HLH patients.

Our study included four patients with malignancy-associated HLH, three had lymphoma, and one had leukemia. Proteomic analysis showed that the expression levels of FCGR2A and CD163 were higher in these patients than in patients with infection-associated HLH. Both FCGR2A and CD163 have been linked to lymphoma and leukemia; they have been studied as diagnostic and prognostic biomarkers [25–27]. One study involving patients with sepsis and features of HLH showed that soluble CD163 (sCD163) could serve as a differential biomarker for sepsis-associated HLH versus sepsis [28]. Gao et al. [29] reported that the serum levels of sCD163 were higher in patients with macrophage activation syndrome (MAS) than in patients with primary HLH. Our study did not include patients with primary HLH or MAS. Because the evidence for FCGR2A and sCD163 as potential biomarkers to distinguish different types of HLH was generated from studies that only included certain types of HLH, validation studies should include all types of HLH to produce definitive conclusions.

A key strength of this study was the approach used to select comparison groups. We chose the comparison groups based on clinical needs. Our findings regarding the proteomic profiles of HLH versus different disease groups contribute to a better understanding of HLH pathophysiology and will serve as a reference for future biomarker studies. Additionally, our proteomic analysis utilized data-driven approaches, offering the opportunity to discover novel biomarkers and pathways with benefits for future studies.

Although this was the first study to investigate the plasma proteomic profile of pediatric HLH, it had several limitations. First, we did not include patients with primary HLH or MAS because these two conditions were relatively rare at our study sites; thus, a proteomic analysis of these conditions would have been underpowered. Second, because MS is not an ideal technique for quantifying cytokines, most of which have low molecular weight and provide few peptides for MS detection [30, 31], we could not validate previous findings regarding the diagnostic value of cytokines for HLH (e.g., IFN-γ, CXCL9, IL6, and IL-10 [4, 8]). Additionally, because of the rarity of HLH, we only validated our findings regarding LCP1 and VCAM1 among six patients with HLH during the study period. Other pathways and biomarkers of interest merit further examination in future studies. For potential biomarkers, it is important to test whether they can identify HLH before the clinical fulfillment of the current diagnostic criteria, which would promote early diagnosis. Moreover, proteomics represents only one level of analysis. Multiomics approaches are needed to gain a comprehensive understanding of the biological changes underlying HLH onset. These approaches constitute a future research direction.

## Conclusion

Our proteomic analysis revealed that HLH is characterized by alterations in pathways involving complement and coagulation cascades, neutrophil extracellular trap formation, and platelet activation. LCP1 emerged as a promising diagnostic marker for HLH. Through comparison with multiple control groups, this study provided a proteomic profile of HLH for researchers pursuing a better understanding of this condition.

### Supplementary information

Supplementary file 1 Supplementary information 1 Supplementary Methods (DOCX 18 kb)

Supplementary file 2 Supplementary information 2 Table S1. Demographic and clinical characteristics of HLH subgroups, Table S2. Proteins with P values of < 0.05 in the comparison between HLH and non-HLH, Table S3. DEPs identified from the comparison between HLH and sepsis, Table S4. DEPs identified from the comparison between HLH and healthy controls, Table S5. DEPs identified from the comparison between HLH and patients from the PICU without infection, Table S6. DEPs identified from the comparison between EBV-HLH and EBV non-HLH (DOCX 66 kb)

Supplementary file 3 Supplementary information 3 Figure S1. Quality control results of indexed retention time/retention time, Figure S2. Correlations between proteomic quantification of FGA, FGG, and clinically tested fibrinogen levels (DOCX 261 kb)

## Data Availability

The dataset used and analyzed during the current study is available from the corresponding author upon reasonable request.

## Abbreviations

ACTB: actin, cytoplasmic 1
ACTG1: actin, cytoplasmic 2
ALB: albumin
AUC: area under the curve
BH: Benjamini-Hochberg
C1QB: complement C1q subcomponent subunit B
CD163: scavenger receptor cysteine-rich type 1 protein M130
CRP: C-reactive protein
DEP: differentially expressed protein
DIA: data-independent acquisition
EBV: Epstein-Barr virus
ECM1: extracellular matrix protein 1
FCGR2A: low affinity immunoglobulin gamma Fc region receptor II-a
FCGR3A: CD16a antigen
FGB: fibrinogen beta chain
FGG: fibrinogen gamma chain
HBG1: hemoglobin subunit gamma-1
HLH: hemophagocytic lymphohistiocytosis
HP: haptoglobin
HPR: haptoglobin-related protein
HSPA8: heat shock cognate 71 kDa protein
IGHV4-4: immunoglobulin heavy variable 4-4
KEGG: Kyoto Encyclopedia of Genes and Genomes
LCP1: lymphocyte cytosolic protein 1, plastin-2
LGALS3BP: galectin-3-binding protein
MODS: multiple organ dysfunction syndrome
MS: mass spectrometry
MSN: moesin
PICU: pediatric intensive care unit
PLG: plasminogen
PRM: parallel reaction monitoring
ROC: receiver operating characteristic
SAA1: serum amyloid A-1 protein
SAA4: serum amyloid A-4 protein
SDK1: protein sidekick-1
VCAM1: vascular cell adhesion protein 1
