# Supplementary material for: Proteomic Analysis of Pediatric Hemophagocytic Lymphohistiocytosis: a Comparative Study with Healthy Controls, Sepsis, Critical Ill, and Active Epstein-Barr virus Infection to Identify Altered Pathways and Candidate Biomarkers
Source: J Clin Immunol. 2023 Aug 31;43(8):1997–2010. doi: 10.1007/s10875-023-01573-w (PMC10661879; doi:10.1007/s10875-023-01573-w)
Supplement: Supplementary file 1 — Supplementary information 1 Supplementary Methods (DOCX 18 kb) [file 10875_2023_1573_MOESM1_ESM.docx]

**Supplementary File 1**

**Methods**

*Data-independent acquisition (DIA)-based proteomic analysis*

DIA-based quantitative proteomic analysis (Shanghai Biotree Co., Ltd. China) was performed for all 5 groups of plasma samples. Plasma samples were prepared for mass spectrometry (MS) as described [1]. For each sample, 2 μg of trypsin-digested peptides were subjected to nano-UHPLC-MS/MS analysis. DIA data were analyzed using the Spectronaut default parameter (BGS Factory Settings). The MS/MS spectra from each run were searched against the species-level UniProt FASTA databases (Homo sapiens-2021-8, reviewed entries 20387). The raw data were processed using SpectroMine (version 2.5.201125.47784; Biognosys AG) software. The search criteria were as follows: tryptic digestion, 2 missed cleavages allowed, carbamidomethyl (C) set as a fixed modification, and oxidation (M) and acetyl (protein N-term) set as variable modifications. Peptide identification was carried out with an initial precursor ion mass deviation of up to 10ppm and a fragment mass deviation of 0.02 Da. For protein identification, the false discovery rate (FDR) was set at 0.01 for both peptide spectral matches (PSMs) and peptide levels. Spectronaut was automatically calibrated and all MS1s that meet the screening criteria are used to calculate the expression levels, and all interfering fragments are excluded except for less than three fragments. Quantity MS-level was set at MS2. Other parameters were set as default.

*Pathway enrichment analysis and network visualization*

The network of enriched functions and pathways was visualized using Cytoscape (version 3.8.2) with the ClueGO and CluePedia plugins. The Kyoto Encyclopedia of Genes and Genomes (KEGG) analysis, performed using clusterProfiler, was employed for pathway enrichment analysis in the comparison between HLH and control groups. An FDR value of 0.05 was utilized as the cutoff.

**Reference**

1. Geyer PE, Kulak NA, Pichler G, Holdt LM, Teupser D, Mann M. Plasma Proteome Profiling to Assess Human Health and Disease. Cell systems. 2016;2(3):185-95. doi:10.1016/j.cels.2016.02.015.
