# Supplementary material for: Proteomic Analysis of Pediatric Hemophagocytic Lymphohistiocytosis: a Comparative Study with Healthy Controls, Sepsis, Critical Ill, and Active Epstein-Barr virus Infection to Identify Altered Pathways and Candidate Biomarkers
Source: J Clin Immunol. 2023 Aug 31;43(8):1997–2010. doi: 10.1007/s10875-023-01573-w (PMC10661879; doi:10.1007/s10875-023-01573-w)
Supplement: Supplementary file 2 — Supplementary information 2 Table S1. Demographic and clinical characteristics of HLH subgroups, Table S2. Proteins with P values of < 0.05 in the comparison between HLH and non-HLH, Table S3. DEPs identified from the comparison between HLH and sepsis, Table S4. DEPs identified from the comparison between HLH and healthy controls, Table S5. DEPs identified from the comparison between HLH and patients from the PICU without infection, Table S6. DEPs identified from the comparison between EBV-HLH and EBV non-HLH (DOCX 66 kb) [file 10875_2023_1573_MOESM2_ESM.docx]

**Supplementary File 2**

**Supplementary Tables**

**Table S1. Demographic and clinical characteristics of the HLH subgroups**

| Variable | EBV-HLH  (n=20) | HLH-other infection  (n=9) | HLH-malignancy  (n=4) | *P* _between HLH subgroups_ | | |
| --- | --- | --- | --- | --- | --- | --- |
|  |  |  |  | _EBV vs other infection_ | _EBV vs malignancy_ | _Other infection vs malignancy_ |
| Age, year | 2.0(1.0, 3.0) | 0.0(0.0, 1.0) | 0 (0, 3.5) | 0.0318 | 0.1534 | 0.7245 |
| Sex |  |  |  |  |  |  |
| Female | 11 (55.0) | 6 (66.7) | 2 (50.0) | 0.6942 | 1.0000 | 1.0000 |
| Male | 9 (45.0) | 3 (33.3) | 2 (50.0) |  |  |  |
| Clinical outcome at hospital discharge |  |  |  |  |  |  |
| Recovery/improvement | **15 (75.0)** | 4 (44.4) | **0** | 0.2047 | **0.0119** | 0.2280 |
| Non-recovery/death | **5 (25.0)** | 5 (55.6) | **4 ( 100)** |  |  |  |
| **Complications during hospitalization** | | | | | | |
| DIC |  |  |  |  |  |  |
| No | 17 (85.0) | 7 (77.8) | 2 (50.0) | 0.6328 | 0.1793 | 0.5301 |
| Yes | 3 (15.0) | 2 (22.2) | 2 (50.0) |  |  |  |
| Shock |  |  |  |  |  |  |
| No | 17 (85.0) | 8 (88.9) | 2 (50.0) | 1.0000 | 0.1793 | 0.2028 |
| Yes | 3 (15.0) | 1 (11.1) | 2 (50.0) |  |  |  |
| Heart failure |  |  |  |  |  |  |
| No | **20 ( 100)** | 8 (88.9) | **1 (25.0)** | 0.3103 | **0.0020** | 0.0517 |
| Yes | **0** | 1 (11.1) | **3 (75.0)** |  |  |  |
| AKI |  |  |  |  |  |  |
| No | 19 (95.0) | 8 (88.9) | 3 (75.0) | 0.5320 | 0.3116 | 1.0000 |
| Yes | 1 ( 5.0) | 1 (11.1) | 1 (25.0) |  |  |  |
| Respiratory failure |  |  |  |  |  |  |
| No | 14 (70.0) | 2 (22.2) | 1 (25.0) | 0.0405 | 0.1304 | 1.0000 |
| Yes | 6 (30.0) | 7 (77.8) | 3 (75.0) |  |  |  |
| ARDS |  |  |  |  |  |  |
| No | 20 ( 100) | 6 (66.7) | 4 ( 100) | 0.0230 | - | 0.4965 |
| Yes | 0 | 3 (33.3) | 0 |  |  |  |
| MODS |  |  |  |  |  |  |
| No | 12 (60.0) | 1 (11.1) | 1 (25.0) | 0.0200 | 0.3002 | 1.0000 |
| Yes | 8 (40.0) | 8 (88.9) | 3 (75.0) |  |  |  |
| **Laboratory tests during hospital admission** | | | | | | |
| Hemoglobin, g/L | 86.50 (71.5, 94.0) | 97.0 (78.0, 114.0) | 100.50 (90.5, 106.5) | 0.2677 | 0.1518 | 1.0000 |
| Neutrophil count, ×10^9^/L | **0.92 (0.39, 1.33)** | **1.94 (1.81, 2.51)** | 2.91 (1.02, 6.57) | **0.0117** | 0.0687 | 1.0000 |
| Platelet count, ×10^9^/L | **53.5 (34.0, 73.5)** | **138.0 (85.0, 281.0)** | 38.5 (22.5, 64.5) | **0.0133** | 0.3938 | 0.0538 |
| White blood cell count, ×10^9^/L | 2.06 (1.25, 2.33) | 3.85 (2.49, 9.58) | 8.79 (2.60, 14.97) | 0.0241 | 0.0624 | 0.6605 |
| Lymphocyte count, ×10^9^/L | 0.98 (0.06, 1.47) | 1.21 (0.58, 3.91) | 3.31 (1.32, 5.30) | 0.2592 | 0.1565 | 0.4642 |
| C-reactive protein, , mg/L | 10.88 (2.04, 34.99) | 16.90 (4.28, 34.52) | 29.56 (23.66, 35.46) | 0.9405 | 0.3020 | 0.4642 |

AKI, acute kidney injury. ARDS, acute respiratory distress syndrome. DIC, disseminated intravascular coagulation. EBV, Epstein-Barr Virus. MODS, multiple organ dysfunction syndrome. HLH, hemophagocytic lymphohistiocytosis. PICU, pediatric intensive care unit.

Values were presented as n (%) or median (quartile 1, quartile 3).

Bold values were statistically significant (P<0.0167) after Bonferroni correction for multiple comparisons (α’= 0.05/3=0.0167).

**Table S2. Proteins with P values of < 0.05 in the comparison between HLH and non-HLH**

| Gene Names | P value | Adjusted P value | Fold change | log2fold | abs_lg2_fd |
| --- | --- | --- | --- | --- | --- |
| H3C1 | <0.0001 | 0.0001 | 10.444 | 3.385 | 3.385 |
| SDK1 | 0.0193 | 0.0899 | 0.124 | -3.011 | 3.011 |
| RAC2 | <0.0001 | 0.0002 | 7.413 | 2.890 | 2.890 |
| ERN1 | <0.0001 | <0.0001 | 7.359 | 2.880 | 2.880 |
| H2AC4 | 0.0005 | 0.0039 | 6.892 | 2.785 | 2.785 |
| ELANE | 0.0085 | 0.0458 | 6.040 | 2.594 | 2.594 |
| H2AX | <0.0001 | <0.0001 | 5.852 | 2.549 | 2.549 |
| S100A8 | <0.0001 | 0.0005 | 5.514 | 2.463 | 2.463 |
| MPO | 0.0004 | 0.0032 | 4.955 | 2.309 | 2.309 |
| H4C1 | <0.0001 | 0.0001 | 4.921 | 2.299 | 2.299 |
| H2BC11 | <0.0001 | 0.0001 | 4.181 | 2.064 | 2.064 |
| PRTN3 | 0.0005 | 0.0039 | 3.884 | 1.958 | 1.958 |
| ACTB | <0.0001 | <0.0001 | 3.850 | 1.945 | 1.945 |
| GPNMB | 0.0003 | 0.0022 | 3.817 | 1.932 | 1.932 |
| FCGR3A | <0.0001 | <0.0001 | 3.807 | 1.928 | 1.928 |
| ACTG1 | <0.0001 | <0.0001 | 3.686 | 1.882 | 1.882 |
| AZU1 | 0.0281 | 0.1171 | 3.583 | 1.841 | 1.841 |
| NACA | 0.0285 | 0.1172 | 3.555 | 1.830 | 1.830 |
| LCP1 | <0.0001 | <0.0001 | 3.542 | 1.825 | 1.825 |
| PF4V1 | 0.0045 | 0.0266 | 0.344 | -1.541 | 1.541 |
| IGLV5-37 | 0.0310 | 0.1253 | 2.839 | 1.506 | 1.506 |
| ACTA2 | <0.0001 | <0.0001 | 2.835 | 1.504 | 1.504 |
| PSMA4 | <0.0001 | 0.0004 | 2.817 | 1.494 | 1.494 |
| PNLIPRP1 | 0.0190 | 0.0898 | 2.812 | 1.491 | 1.491 |
| PPBP | 0.0005 | 0.0044 | 0.357 | -1.486 | 1.486 |
| CORO1A | <0.0001 | <0.0001 | 2.596 | 1.376 | 1.376 |
| PSMB2 | <0.0001 | 0.0001 | 2.535 | 1.342 | 1.342 |
| FCGR2A | <0.0001 | 0.0003 | 2.506 | 1.326 | 1.326 |
| APOC1 | <0.0001 | <0.0001 | 0.409 | -1.290 | 1.290 |
| SELENOP | <0.0001 | <0.0001 | 0.412 | -1.280 | 1.280 |
| H2BC12 | <0.0001 | 0.0002 | 2.426 | 1.279 | 1.279 |
| S100A9 | 0.0002 | 0.0017 | 2.357 | 1.237 | 1.237 |
| IGHV2-70 | 0.0206 | 0.0944 | 2.352 | 1.234 | 1.234 |
| DEFA3 | <0.0001 | <0.0001 | 2.340 | 1.226 | 1.226 |
| CTSG | 0.0238 | 0.1040 | 2.339 | 1.226 | 1.226 |
| CD163 | <0.0001 | <0.0001 | 2.337 | 1.225 | 1.225 |
| EEF1G | 0.0040 | 0.0240 | 2.268 | 1.182 | 1.182 |
| ARPC3 | 0.0093 | 0.0498 | 2.255 | 1.173 | 1.173 |
| IGHV1-69D | 0.0193 | 0.0899 | 0.447 | -1.162 | 1.162 |
| PIGR | 0.0010 | 0.0073 | 2.220 | 1.151 | 1.151 |
| PSMB9 | 0.0024 | 0.0158 | 2.185 | 1.128 | 1.128 |
| ARPC5 | 0.0105 | 0.0553 | 2.176 | 1.122 | 1.122 |
| PSMA6 | 0.0003 | 0.0028 | 2.164 | 1.113 | 1.113 |
| APOA1 | <0.0001 | <0.0001 | 0.466 | -1.103 | 1.103 |
| IGFBP1 | 0.0146 | 0.0729 | 2.131 | 1.092 | 1.092 |
| ICAM1 | <0.0001 | <0.0001 | 2.103 | 1.073 | 1.073 |
| EIF2S3 | 0.0088 | 0.0472 | 2.088 | 1.062 | 1.062 |
| PCBP1 | 0.0028 | 0.0177 | 2.085 | 1.060 | 1.060 |
| VCAM1 | <0.0001 | <0.0001 | 2.057 | 1.041 | 1.041 |
| LUM | <0.0001 | <0.0001 | 0.488 | -1.035 | 1.035 |
| MSN | <0.0001 | <0.0001 | 2.048 | 1.034 | 1.034 |
| FAH | 0.0123 | 0.0624 | 2.041 | 1.029 | 1.029 |
| HLA-A | <0.0001 | <0.0001 | 2.034 | 1.025 | 1.025 |
| IGFALS | 0.0002 | 0.0015 | 0.493 | -1.022 | 1.022 |
| RAC1 | 0.0001 | 0.0010 | 2.026 | 1.018 | 1.018 |
| HSP90AB1 | 0.0002 | 0.0015 | 1.993 | 0.995 | 0.995 |
| ECM1 | <0.0001 | <0.0001 | 0.508 | -0.977 | 0.977 |
| CD14 | <0.0001 | <0.0001 | 1.965 | 0.974 | 0.974 |
| B2M | 0.0008 | 0.0062 | 1.965 | 0.974 | 0.974 |
| MTHFD1 | 0.0288 | 0.1180 | 1.946 | 0.960 | 0.960 |
| AHSG | <0.0001 | <0.0001 | 0.518 | -0.948 | 0.948 |
| SERPINA5 | <0.0001 | <0.0001 | 0.525 | -0.929 | 0.929 |
| HPR | 0.0007 | 0.0057 | 0.528 | -0.923 | 0.923 |
| IGLC7 | 0.0067 | 0.0371 | 1.885 | 0.915 | 0.915 |
| HSP90AA1 | <0.0001 | 0.0002 | 1.878 | 0.909 | 0.909 |
| LGALS3BP | <0.0001 | <0.0001 | 1.876 | 0.908 | 0.908 |
| IL18BP | <0.0001 | <0.0001 | 1.868 | 0.902 | 0.902 |
| IGKV1D-33 | 0.0426 | 0.1569 | 0.536 | -0.900 | 0.900 |
| A2M | <0.0001 | <0.0001 | 0.536 | -0.899 | 0.899 |
| ROR1 | 0.0001 | 0.0010 | 0.544 | -0.878 | 0.878 |
| VCAN | 0.0002 | 0.0016 | 1.835 | 0.876 | 0.876 |
| ALB | <0.0001 | <0.0001 | 0.545 | -0.875 | 0.875 |
| CFHR4 | 0.0021 | 0.0141 | 0.552 | -0.858 | 0.858 |
| KCTD12 | 0.0004 | 0.0034 | 1.790 | 0.840 | 0.840 |
| TYMP | <0.0001 | <0.0001 | 1.785 | 0.836 | 0.836 |
| CD5L | 0.0003 | 0.0029 | 0.564 | -0.826 | 0.826 |
| COG4 | 0.0331 | 0.1310 | 0.566 | -0.820 | 0.820 |
| FGB | <0.0001 | <0.0001 | 0.567 | -0.818 | 0.818 |
| PSMB3 | 0.0027 | 0.0168 | 1.755 | 0.811 | 0.811 |
| PSMA3 | <0.0001 | 0.0001 | 1.742 | 0.801 | 0.801 |
| APOH | <0.0001 | <0.0001 | 0.575 | -0.799 | 0.799 |
| PSAP | 0.0171 | 0.0823 | 1.732 | 0.793 | 0.793 |
| FGG | <0.0001 | <0.0001 | 0.577 | -0.792 | 0.792 |
| PSMB4 | <0.0001 | <0.0001 | 1.731 | 0.792 | 0.792 |
| ADA2 | <0.0001 | <0.0001 | 1.731 | 0.792 | 0.792 |
| IGFBP3 | 0.0008 | 0.0058 | 0.578 | -0.791 | 0.791 |
| HP | 0.0156 | 0.0767 | 0.579 | -0.789 | 0.789 |
| APOM | <0.0001 | <0.0001 | 0.581 | -0.784 | 0.784 |
| FGA | <0.0001 | <0.0001 | 0.581 | -0.783 | 0.783 |
| ARPC1B | 0.0244 | 0.1057 | 1.717 | 0.780 | 0.780 |
| WARS1 | <0.0001 | <0.0001 | 1.712 | 0.776 | 0.776 |
| PRDX1 | 0.0257 | 0.1097 | 1.702 | 0.767 | 0.767 |
| SAA4 | <0.0001 | <0.0001 | 0.588 | -0.767 | 0.767 |
| HRG | <0.0001 | <0.0001 | 0.588 | -0.766 | 0.766 |
| TF | <0.0001 | <0.0001 | 0.590 | -0.761 | 0.761 |
| PLA1A | 0.0012 | 0.0088 | 1.670 | 0.740 | 0.740 |
| ARPC4 | 0.0005 | 0.0042 | 1.665 | 0.735 | 0.735 |
| ITIH2 | <0.0001 | <0.0001 | 0.606 | -0.722 | 0.722 |
| GSN | <0.0001 | <0.0001 | 0.608 | -0.719 | 0.719 |
| TUBA1A | 0.0024 | 0.0156 | 1.643 | 0.716 | 0.716 |
| RHOG | 0.0251 | 0.1081 | 1.642 | 0.716 | 0.716 |
| YWHAB | 0.0069 | 0.0379 | 1.639 | 0.713 | 0.713 |
| PI16 | 0.0112 | 0.0580 | 0.611 | -0.711 | 0.711 |
| EEF2 | <0.0001 | 0.0001 | 1.626 | 0.701 | 0.701 |
| APOA4 | 0.0001 | 0.0006 | 0.621 | -0.688 | 0.688 |
| CPB2 | <0.0001 | 0.0001 | 0.623 | -0.682 | 0.682 |
| TTR | <0.0001 | <0.0001 | 0.625 | -0.679 | 0.679 |
| SERPINA4 | <0.0001 | 0.0003 | 0.626 | -0.676 | 0.676 |
| CLEC3B | <0.0001 | <0.0001 | 0.626 | -0.675 | 0.675 |
| HGFAC | <0.0001 | <0.0001 | 0.626 | -0.675 | 0.675 |
| VIM | 0.0001 | 0.0014 | 1.593 | 0.672 | 0.672 |
| BCHE | <0.0001 | <0.0001 | 0.628 | -0.672 | 0.672 |
| SIGLEC14 | 0.0007 | 0.0051 | 1.591 | 0.670 | 0.670 |
| IGLV6-57 | 0.0283 | 0.1172 | 0.630 | -0.666 | 0.666 |
| CAPZA1 | 0.0112 | 0.0580 | 1.582 | 0.661 | 0.661 |
| EEF1D | 0.0079 | 0.0433 | 1.576 | 0.656 | 0.656 |
| IGHV3-43 | 0.0434 | 0.1587 | 0.636 | -0.653 | 0.653 |
| PON1 | <0.0001 | <0.0001 | 0.637 | -0.651 | 0.651 |
| HPRT1 | 0.0015 | 0.0111 | 1.551 | 0.633 | 0.633 |
| CSF1R | <0.0001 | <0.0001 | 1.546 | 0.629 | 0.629 |
| RBP4 | 0.0002 | 0.0020 | 0.649 | -0.624 | 0.624 |
| EEF1A1 | 0.0020 | 0.0138 | 1.539 | 0.622 | 0.622 |
| YWHAG | 0.0025 | 0.0164 | 1.537 | 0.620 | 0.620 |
| CBLN4 | 0.0198 | 0.0918 | 0.654 | -0.612 | 0.612 |
| GPLD1 | <0.0001 | <0.0001 | 0.656 | -0.608 | 0.608 |
| IGKV2D-24 | 0.0245 | 0.1058 | 0.659 | -0.602 | 0.602 |
| ADIPOQ | 0.0276 | 0.1158 | 0.665 | -0.589 | 0.589 |
| F2 | <0.0001 | <0.0001 | 0.667 | -0.585 | 0.585 |
| USP5 | 0.0045 | 0.0266 | 1.494 | 0.579 | 0.579 |
| RAB5C | 0.0260 | 0.1097 | 1.493 | 0.578 | 0.578 |
| RPSA | 0.0163 | 0.0791 | 1.489 | 0.574 | 0.574 |
| ATRN | <0.0001 | <0.0001 | 0.672 | -0.572 | 0.572 |
| IGF2 | <0.0001 | <0.0001 | 0.674 | -0.569 | 0.569 |
| CETP | <0.0001 | <0.0001 | 0.675 | -0.568 | 0.568 |
| HSPA1A | 0.0036 | 0.0218 | 1.481 | 0.567 | 0.567 |
| CTSD | <0.0001 | 0.0001 | 1.478 | 0.564 | 0.564 |
| GSS | 0.0001 | 0.0013 | 1.472 | 0.558 | 0.558 |
| SERPIND1 | <0.0001 | <0.0001 | 0.680 | -0.556 | 0.556 |
| APOA2 | <0.0001 | <0.0001 | 0.681 | -0.554 | 0.554 |
| AFM | <0.0001 | <0.0001 | 0.681 | -0.554 | 0.554 |
| GP1BA | 0.0042 | 0.0251 | 1.465 | 0.551 | 0.551 |
| PSMA5 | <0.0001 | <0.0001 | 1.462 | 0.548 | 0.548 |
| IGHV3-15 | 0.0294 | 0.1202 | 0.685 | -0.545 | 0.545 |
| SNRPB | 0.0048 | 0.0276 | 1.458 | 0.544 | 0.544 |
| ACSL1 | 0.0027 | 0.0172 | 1.455 | 0.541 | 0.541 |
| LBP | 0.0058 | 0.0330 | 1.451 | 0.538 | 0.538 |
| APMAP | 0.0003 | 0.0028 | 0.690 | -0.536 | 0.536 |
| PSMB10 | <0.0001 | 0.0002 | 1.448 | 0.534 | 0.534 |
| S100P | 0.0447 | 0.1623 | 1.446 | 0.532 | 0.532 |
| ISG15 | <0.0001 | 0.0003 | 1.444 | 0.530 | 0.530 |
| KLKB1 | <0.0001 | <0.0001 | 0.693 | -0.529 | 0.529 |
| MAT2A | 0.0001 | 0.0006 | 1.442 | 0.528 | 0.528 |
| CYBB | 0.0112 | 0.0580 | 1.438 | 0.524 | 0.524 |
| TKT | 0.0016 | 0.0112 | 1.434 | 0.520 | 0.520 |
| CD99 | 0.0372 | 0.1416 | 0.702 | -0.511 | 0.511 |
| YWHAZ | <0.0001 | 0.0002 | 1.423 | 0.509 | 0.509 |
| C3 | <0.0001 | <0.0001 | 0.703 | -0.509 | 0.509 |
| YWHAE | 0.0108 | 0.0568 | 1.420 | 0.506 | 0.506 |
| RPS16 | 0.0046 | 0.0266 | 1.420 | 0.506 | 0.506 |
| GAPDH | 0.0002 | 0.0016 | 1.416 | 0.502 | 0.502 |
| ICAM3 | 0.0001 | 0.0010 | 1.416 | 0.502 | 0.502 |
| FUOM | 0.0325 | 0.1299 | 1.412 | 0.498 | 0.498 |
| THBS1 | 0.0110 | 0.0574 | 0.710 | -0.495 | 0.495 |
| TALDO1 | 0.0001 | 0.0006 | 1.404 | 0.489 | 0.489 |
| GNPDA1 | <0.0001 | <0.0001 | 1.402 | 0.487 | 0.487 |
| PPP2CB | 0.0315 | 0.1264 | 1.400 | 0.485 | 0.485 |
| PROS1 | <0.0001 | <0.0001 | 0.715 | -0.485 | 0.485 |
| GC | <0.0001 | <0.0001 | 0.716 | -0.482 | 0.482 |
| C8B | <0.0001 | <0.0001 | 0.717 | -0.480 | 0.480 |
| HSPA8 | 0.0002 | 0.0017 | 1.394 | 0.480 | 0.480 |
| MVB12B | 0.0326 | 0.1300 | 0.719 | -0.476 | 0.476 |
| C8A | <0.0001 | <0.0001 | 0.719 | -0.475 | 0.475 |
| NID1 | <0.0001 | <0.0001 | 1.387 | 0.472 | 0.472 |
| CFHR1 | <0.0001 | 0.0001 | 0.723 | -0.468 | 0.468 |
| OSMR | 0.0109 | 0.0571 | 1.381 | 0.465 | 0.465 |
| PGLYRP2 | <0.0001 | <0.0001 | 0.725 | -0.465 | 0.465 |
| OAF | <0.0001 | <0.0001 | 1.378 | 0.463 | 0.463 |
| PLG | <0.0001 | <0.0001 | 0.730 | -0.455 | 0.455 |
| GOLM1 | <0.0001 | <0.0001 | 1.366 | 0.450 | 0.450 |
| RBBP4 | 0.0016 | 0.0113 | 1.362 | 0.446 | 0.446 |
| SERPINE1 | 0.0236 | 0.1037 | 1.361 | 0.445 | 0.445 |
| NAGK | <0.0001 | <0.0001 | 1.360 | 0.444 | 0.444 |
| GPX3 | 0.0498 | 0.1758 | 1.359 | 0.443 | 0.443 |
| MRC1 | <0.0001 | <0.0001 | 1.358 | 0.442 | 0.442 |
| SERPINB1 | <0.0001 | <0.0001 | 1.356 | 0.439 | 0.439 |
| CTSS | <0.0001 | 0.0001 | 1.353 | 0.436 | 0.436 |
| CAPNS1 | 0.0003 | 0.0030 | 1.353 | 0.436 | 0.436 |
| ITIH1 | <0.0001 | <0.0001 | 0.740 | -0.435 | 0.435 |
| C8G | <0.0001 | <0.0001 | 0.740 | -0.435 | 0.435 |
| TUBA4A | 0.0021 | 0.0139 | 1.351 | 0.434 | 0.434 |
| ASAH1 | 0.0197 | 0.0916 | 1.346 | 0.428 | 0.428 |
| PFN1 | 0.0371 | 0.1416 | 1.343 | 0.426 | 0.426 |
| S100A12 | 0.0001 | 0.0008 | 1.343 | 0.425 | 0.425 |
| IGFBP2 | 0.0065 | 0.0363 | 1.340 | 0.422 | 0.422 |
| RHOA | 0.0006 | 0.0049 | 1.338 | 0.420 | 0.420 |
| SHBG | 0.0078 | 0.0427 | 0.748 | -0.420 | 0.420 |
| APOD | <0.0001 | 0.0002 | 0.748 | -0.419 | 0.419 |
| MACROH2A1 | 0.0174 | 0.0834 | 1.336 | 0.418 | 0.418 |
| LRG1 | 0.0026 | 0.0164 | 1.336 | 0.418 | 0.418 |
| NUCB1 | <0.0001 | <0.0001 | 1.336 | 0.418 | 0.418 |
| C4BPB | <0.0001 | <0.0001 | 0.750 | -0.416 | 0.416 |
| SERPINA6 | <0.0001 | <0.0001 | 0.752 | -0.412 | 0.412 |
| LILRA3 | 0.0001 | 0.0009 | 1.326 | 0.408 | 0.408 |
| GPI | 0.0116 | 0.0592 | 1.326 | 0.407 | 0.407 |
| RNH1 | <0.0001 | 0.0002 | 1.324 | 0.405 | 0.405 |
| KRT2 | 0.0380 | 0.1439 | 0.756 | -0.404 | 0.404 |
| MARCO | 0.0030 | 0.0187 | 1.319 | 0.399 | 0.399 |
| PSMA7 | <0.0001 | 0.0001 | 1.317 | 0.397 | 0.397 |
| FOLR2 | 0.0025 | 0.0160 | 1.315 | 0.395 | 0.395 |
| FN1 | 0.0020 | 0.0138 | 0.761 | -0.394 | 0.394 |
| C6 | <0.0001 | <0.0001 | 0.761 | -0.393 | 0.393 |
| PDCD6 | 0.0121 | 0.0619 | 1.312 | 0.392 | 0.392 |
| HK3 | <0.0001 | <0.0001 | 1.312 | 0.392 | 0.392 |
| KRT10 | 0.0259 | 0.1097 | 0.763 | -0.390 | 0.390 |
| PSMA1 | <0.0001 | <0.0001 | 1.308 | 0.388 | 0.388 |
| LCAT | <0.0001 | <0.0001 | 0.765 | -0.387 | 0.387 |
| TFG | 0.0244 | 0.1057 | 1.306 | 0.385 | 0.385 |
| CLU | <0.0001 | <0.0001 | 0.766 | -0.384 | 0.384 |
| SERPINC1 | <0.0001 | 0.0002 | 0.767 | -0.383 | 0.383 |
| CORO1B | 0.0210 | 0.0951 | 1.304 | 0.383 | 0.383 |
| VAT1 | 0.0008 | 0.0058 | 1.303 | 0.382 | 0.382 |
| WDR1 | 0.0278 | 0.1164 | 1.303 | 0.381 | 0.381 |
| CD44 | 0.0104 | 0.0550 | 1.300 | 0.378 | 0.378 |
| CTSZ | 0.0079 | 0.0433 | 1.297 | 0.375 | 0.375 |
| AZGP1 | 0.0007 | 0.0056 | 0.774 | -0.369 | 0.369 |
| KRT1 | 0.0411 | 0.1527 | 0.780 | -0.359 | 0.359 |
| ARPC2 | 0.0379 | 0.1437 | 1.280 | 0.356 | 0.356 |
| LTA4H | <0.0001 | <0.0001 | 1.279 | 0.355 | 0.355 |
| LDHA | 0.0394 | 0.1479 | 1.278 | 0.354 | 0.354 |
| GSR | <0.0001 | <0.0001 | 1.277 | 0.352 | 0.352 |
| POR | 0.0400 | 0.1494 | 1.270 | 0.344 | 0.344 |
| VWF | <0.0001 | 0.0002 | 1.267 | 0.341 | 0.341 |
| VTN | 0.0001 | 0.0011 | 0.792 | -0.336 | 0.336 |
| MNDA | 0.0049 | 0.0281 | 1.260 | 0.334 | 0.334 |
| SPP2 | 0.0389 | 0.1464 | 0.794 | -0.332 | 0.332 |
| CAPZA2 | 0.0064 | 0.0361 | 1.258 | 0.331 | 0.331 |
| CALM1 | 0.0020 | 0.0138 | 1.255 | 0.328 | 0.328 |
| CDH5 | 0.0017 | 0.0122 | 0.798 | -0.326 | 0.326 |
| PCSK9 | <0.0001 | <0.0001 | 1.252 | 0.325 | 0.325 |
| CFH | <0.0001 | <0.0001 | 0.801 | -0.321 | 0.321 |
| APOB | 0.0002 | 0.0015 | 0.803 | -0.317 | 0.317 |
| FUCA1 | <0.0001 | 0.0001 | 1.246 | 0.317 | 0.317 |
| HPX | 0.0020 | 0.0138 | 0.803 | -0.316 | 0.316 |
| F13B | <0.0001 | <0.0001 | 0.806 | -0.312 | 0.312 |
| PSME1 | 0.0491 | 0.1746 | 1.241 | 0.312 | 0.312 |
| SIL1 | 0.0226 | 0.1004 | 1.238 | 0.308 | 0.308 |
| STAT1 | 0.0228 | 0.1010 | 1.236 | 0.306 | 0.306 |
| KNG1 | 0.0012 | 0.0089 | 0.811 | -0.302 | 0.302 |
| H2AZ1 | 0.0213 | 0.0963 | 1.231 | 0.300 | 0.300 |
| RDX | 0.0002 | 0.0014 | 1.231 | 0.299 | 0.299 |
| ANPEP | 0.0001 | 0.0006 | 1.225 | 0.293 | 0.293 |
| RAB8A | 0.0163 | 0.0791 | 1.223 | 0.290 | 0.290 |
| F13A1 | 0.0034 | 0.0211 | 0.819 | -0.287 | 0.287 |
| CORO1C | 0.0314 | 0.1264 | 1.220 | 0.287 | 0.287 |
| AXL | 0.0025 | 0.0162 | 1.220 | 0.287 | 0.287 |
| APOF | 0.0275 | 0.1155 | 0.822 | -0.283 | 0.283 |
| CFB | 0.0017 | 0.0122 | 0.823 | -0.282 | 0.282 |
| PAM | 0.0199 | 0.0919 | 1.213 | 0.279 | 0.279 |
| F9 | 0.0007 | 0.0053 | 0.824 | -0.279 | 0.279 |
| PSMA2 | 0.0017 | 0.0120 | 1.212 | 0.277 | 0.277 |
| GGH | 0.0010 | 0.0076 | 1.209 | 0.274 | 0.274 |
| CFI | <0.0001 | <0.0001 | 0.828 | -0.273 | 0.273 |
| C4BPA | 0.0002 | 0.0015 | 0.828 | -0.272 | 0.272 |
| ARHGDIB | 0.0130 | 0.0654 | 1.208 | 0.272 | 0.272 |
| MYL6 | 0.0337 | 0.1321 | 1.207 | 0.271 | 0.271 |
| IL1R2 | 0.0038 | 0.0231 | 1.205 | 0.269 | 0.269 |
| PCBP2 | 0.0053 | 0.0300 | 1.204 | 0.268 | 0.268 |
| FCN2 | 0.0005 | 0.0042 | 0.832 | -0.265 | 0.265 |
| PSMB6 | 0.0492 | 0.1746 | 1.199 | 0.262 | 0.262 |
| RAB10 | 0.0024 | 0.0156 | 1.199 | 0.262 | 0.262 |
| PLTP | 0.0026 | 0.0168 | 1.198 | 0.261 | 0.261 |
| SEMA4B | 0.0004 | 0.0032 | 1.197 | 0.260 | 0.260 |
| MAT2B | 0.0007 | 0.0052 | 1.197 | 0.259 | 0.259 |
| PGAM1 | 0.0014 | 0.0101 | 1.197 | 0.259 | 0.259 |
| HABP2 | 0.0001 | 0.0007 | 0.836 | -0.259 | 0.259 |
| CTSC | 0.0156 | 0.0767 | 1.194 | 0.256 | 0.256 |
| EFHD2 | 0.0230 | 0.1015 | 1.194 | 0.255 | 0.255 |
| LDHB | <0.0001 | 0.0003 | 1.193 | 0.255 | 0.255 |
| PROC | 0.0021 | 0.0141 | 0.839 | -0.253 | 0.253 |
| CPN1 | 0.0002 | 0.0016 | 0.840 | -0.252 | 0.252 |
| VCL | <0.0001 | <0.0001 | 1.191 | 0.252 | 0.252 |
| TGFBI | 0.0001 | 0.0012 | 1.189 | 0.250 | 0.250 |
| AMBP | 0.0002 | 0.0018 | 0.841 | -0.250 | 0.250 |
| GNB2 | 0.0030 | 0.0188 | 1.188 | 0.248 | 0.248 |
| PGC | 0.0285 | 0.1172 | 1.187 | 0.248 | 0.248 |
| COLEC10 | 0.0043 | 0.0256 | 0.845 | -0.243 | 0.243 |
| CRTAC1 | 0.0007 | 0.0053 | 0.846 | -0.242 | 0.242 |
| SERPINB8 | 0.0025 | 0.0160 | 1.181 | 0.240 | 0.240 |
| LMAN2 | 0.0001 | 0.0012 | 1.180 | 0.239 | 0.239 |
| SERPINF2 | 0.0001 | 0.0010 | 0.848 | -0.238 | 0.238 |
| EZR | 0.0020 | 0.0138 | 1.177 | 0.235 | 0.235 |
| B4GALT1 | <0.0001 | 0.0004 | 1.176 | 0.234 | 0.234 |
| MASP1 | 0.0003 | 0.0030 | 0.853 | -0.230 | 0.230 |
| TFPI | 0.0167 | 0.0810 | 1.172 | 0.229 | 0.229 |
| TPP1 | 0.0330 | 0.1310 | 1.171 | 0.227 | 0.227 |
| SERPING1 | 0.0055 | 0.0314 | 1.170 | 0.226 | 0.226 |
| PON3 | 0.0038 | 0.0231 | 0.855 | -0.226 | 0.226 |
| FGL1 | 0.0152 | 0.0753 | 1.166 | 0.222 | 0.222 |
| SERPINF1 | 0.0066 | 0.0367 | 0.859 | -0.220 | 0.220 |
| MST1 | 0.0001 | 0.0010 | 0.859 | -0.219 | 0.219 |
| ACTR2 | 0.0064 | 0.0361 | 1.164 | 0.219 | 0.219 |
| PVR | 0.0258 | 0.1097 | 1.163 | 0.218 | 0.218 |
| ILF3 | <0.0001 | <0.0001 | 1.163 | 0.218 | 0.218 |
| PFKL | 0.0329 | 0.1310 | 1.162 | 0.217 | 0.217 |
| ARF3 | 0.0040 | 0.0238 | 1.162 | 0.217 | 0.217 |
| F11 | 0.0135 | 0.0679 | 0.861 | -0.216 | 0.216 |
| C1QTNF3 | 0.0181 | 0.0862 | 0.862 | -0.214 | 0.214 |
| FSTL1 | 0.0222 | 0.0998 | 1.160 | 0.214 | 0.214 |
| HK1 | 0.0100 | 0.0534 | 1.160 | 0.214 | 0.214 |
| GPX1 | 0.0360 | 0.1390 | 1.158 | 0.211 | 0.211 |
| CAPZB | 0.0339 | 0.1326 | 1.152 | 0.204 | 0.204 |
| BTD | 0.0045 | 0.0266 | 0.870 | -0.201 | 0.201 |
| CP | 0.0013 | 0.0092 | 0.870 | -0.201 | 0.201 |
| PCYOX1 | 0.0017 | 0.0119 | 0.872 | -0.197 | 0.197 |
| FGL2 | 0.0001 | 0.0008 | 1.146 | 0.197 | 0.197 |
| ITIH4 | 0.0031 | 0.0193 | 0.874 | -0.194 | 0.194 |
| C5 | 0.0012 | 0.0085 | 0.875 | -0.193 | 0.193 |
| PTPN6 | 0.0441 | 0.1603 | 1.139 | 0.188 | 0.188 |
| GRN | 0.0004 | 0.0034 | 1.137 | 0.185 | 0.185 |
| CAPN1 | 0.0128 | 0.0647 | 1.136 | 0.184 | 0.184 |
| INHBE | 0.0224 | 0.0998 | 1.136 | 0.184 | 0.184 |
| PEPD | 0.0039 | 0.0236 | 1.134 | 0.182 | 0.182 |
| BST1 | 0.0140 | 0.0703 | 1.134 | 0.182 | 0.182 |
| PSMB8 | 0.0045 | 0.0266 | 1.131 | 0.178 | 0.178 |
| PA2G4 | 0.0470 | 0.1688 | 1.130 | 0.176 | 0.176 |
| IGF2R | <0.0001 | <0.0001 | 1.126 | 0.171 | 0.171 |
| FUCA2 | 0.0094 | 0.0500 | 1.125 | 0.170 | 0.170 |
| TXN | 0.0352 | 0.1368 | 1.125 | 0.169 | 0.169 |
| S100A11 | 0.0403 | 0.1500 | 1.124 | 0.169 | 0.169 |
| PGD | 0.0115 | 0.0590 | 1.123 | 0.167 | 0.167 |
| SPARC | 0.0283 | 0.1172 | 0.892 | -0.166 | 0.166 |
| C7 | 0.0303 | 0.1232 | 0.893 | -0.163 | 0.163 |
| MYH9 | 0.0239 | 0.1040 | 1.117 | 0.160 | 0.160 |
| PSMB1 | <0.0001 | <0.0001 | 1.116 | 0.159 | 0.159 |
| ENPP2 | 0.0004 | 0.0034 | 1.115 | 0.157 | 0.157 |
| IGFBP7 | 0.0175 | 0.0837 | 0.898 | -0.156 | 0.156 |
| HYOU1 | 0.0254 | 0.1092 | 1.113 | 0.155 | 0.155 |
| QSOX1 | <0.0001 | 0.0004 | 1.113 | 0.154 | 0.154 |
| CDH1 | 0.0191 | 0.0898 | 1.112 | 0.153 | 0.153 |
| IGFBP5 | 0.0190 | 0.0898 | 0.900 | -0.153 | 0.153 |
| PPP1CA | 0.0295 | 0.1204 | 1.109 | 0.150 | 0.150 |
| APRT | 0.0310 | 0.1253 | 1.107 | 0.147 | 0.147 |
| CFD | 0.0435 | 0.1587 | 0.907 | -0.140 | 0.140 |
| ATIC | 0.0429 | 0.1573 | 1.101 | 0.139 | 0.139 |
| HSPA4 | 0.0051 | 0.0294 | 1.100 | 0.138 | 0.138 |
| HEXB | 0.0369 | 0.1416 | 1.100 | 0.137 | 0.137 |
| TLN1 | 0.0208 | 0.0948 | 1.099 | 0.136 | 0.136 |
| RPIA | 0.0181 | 0.0862 | 1.099 | 0.136 | 0.136 |
| PTPRJ | 0.0256 | 0.1096 | 1.098 | 0.135 | 0.135 |
| A1BG | 0.0451 | 0.1627 | 0.911 | -0.135 | 0.135 |
| PDCD6IP | 0.0354 | 0.1373 | 1.097 | 0.133 | 0.133 |
| HLA-B | 0.0371 | 0.1416 | 1.095 | 0.131 | 0.131 |
| PLA2G7 | 0.0214 | 0.0963 | 1.093 | 0.128 | 0.128 |
| ITLN1 | 0.0397 | 0.1484 | 0.916 | -0.127 | 0.127 |
| SMPDL3A | 0.0474 | 0.1697 | 1.091 | 0.126 | 0.126 |
| ADAMTS13 | 0.0006 | 0.0047 | 0.919 | -0.122 | 0.122 |
| KIT | 0.0337 | 0.1321 | 0.923 | -0.116 | 0.116 |
| IQGAP1 | 0.0223 | 0.0998 | 1.082 | 0.114 | 0.114 |
| UGGT1 | 0.0344 | 0.1342 | 1.080 | 0.111 | 0.111 |
| ISOC2 | 0.0426 | 0.1569 | 0.927 | -0.109 | 0.109 |
| CR2 | 0.0419 | 0.1549 | 0.931 | -0.104 | 0.104 |
| LRP1 | 0.0205 | 0.0941 | 1.074 | 0.103 | 0.103 |
| MAN1A1 | 0.0208 | 0.0948 | 1.074 | 0.103 | 0.103 |
| MINPP1 | 0.0082 | 0.0448 | 1.072 | 0.100 | 0.100 |
| SLC3A2 | 0.0449 | 0.1625 | 1.072 | 0.100 | 0.100 |
| CHL1 | 0.0230 | 0.1015 | 0.935 | -0.097 | 0.097 |
| CAND1 | 0.0464 | 0.1672 | 1.069 | 0.097 | 0.097 |
| MGAT1 | 0.0336 | 0.1321 | 1.067 | 0.093 | 0.093 |
| MAN2A1 | 0.0154 | 0.0761 | 1.065 | 0.090 | 0.090 |
| TNXB | 0.0381 | 0.1440 | 0.943 | -0.085 | 0.085 |
| YWHAH | 0.0372 | 0.1416 | 1.061 | 0.085 | 0.085 |
| IGFBP4 | 0.0498 | 0.1758 | 1.060 | 0.084 | 0.084 |
| HSPG2 | 0.0483 | 0.1723 | 0.944 | -0.083 | 0.083 |
| GDI1 | 0.0158 | 0.0772 | 0.944 | -0.083 | 0.083 |

**Table S3. DEPs identified from the comparison between HLH and sepsis**

| Gene Names | P value | Adjusted P value | Fold change | log2fold | abs_lg2_fd | Fold change rank |
| --- | --- | --- | --- | --- | --- | --- |
| SDK1 | 0.0010 | 0.0305 | 0.071 | -3.821 | 3.821 | 1 |
| SAA1 | 0.0008 | 0.0254 | 0.220 | -2.183 | 2.183 | 2 |
| ACTB | <0.0001 | 0.0011 | 3.405 | 1.768 | 1.768 | 3 |
| CRP | 0.0006 | 0.0216 | 0.296 | -1.757 | 1.757 | 4 |
| ACTG1 | 0.0012 | 0.0337 | 3.317 | 1.730 | 1.730 | 5 |
| FCGR3A | <0.0001 | 0.0012 | 3.199 | 1.678 | 1.678 | 6 |
| LCP1 | <0.0001 | <0.0001 | 3.156 | 1.658 | 1.658 | 7 |
| HP | <0.0001 | 0.0015 | 0.347 | -1.528 | 1.528 | 8 |
| HPR | <0.0001 | 0.0011 | 0.359 | -1.477 | 1.477 | 9 |
| HSP90AB1 | 0.0020 | 0.0465 | 2.059 | 1.042 | 1.042 | 10 |
| FGA | <0.0001 | <0.0001 | 0.499 | -1.002 | 1.002 | 11 |
| HLA-A | 0.0021 | 0.0466 | 1.991 | 0.993 | 0.993 | 12 |
| FGB | <0.0001 | 0.0004 | 0.504 | -0.988 | 0.988 | 13 |
| MSN | 0.0010 | 0.0310 | 1.957 | 0.969 | 0.969 | 14 |
| VCAM1 | <0.0001 | 0.0004 | 1.949 | 0.962 | 0.962 | 15 |
| FGG | <0.0001 | <0.0001 | 0.517 | -0.951 | 0.951 | 16 |
| HSP90AA1 | 0.0003 | 0.0117 | 1.902 | 0.927 | 0.927 | 17 |
| IL18BP | <0.0001 | 0.0024 | 1.851 | 0.888 | 0.888 | 18 |
| TYMP | <0.0001 | 0.0007 | 1.812 | 0.858 | 0.858 | 19 |
| ADA2 | 0.0001 | 0.0043 | 1.750 | 0.807 | 0.807 | 20 |
| WARS1 | <0.0001 | 0.0004 | 1.718 | 0.781 | 0.781 | 21 |
| LGALS3BP | 0.0002 | 0.0107 | 1.680 | 0.748 | 0.748 | 22 |
| SAA4 | 0.0004 | 0.0164 | 0.624 | -0.682 | 0.682 | 23 |
| LAP3 | 0.0009 | 0.0295 | 1.591 | 0.670 | 0.670 | 24 |
| ECM1 | <0.0001 | <0.0001 | 0.632 | -0.662 | 0.662 | 25 |
| APMAP | 0.0016 | 0.0374 | 0.648 | -0.625 | 0.625 | 26 |
| APOA1 | <0.0001 | 0.0015 | 0.650 | -0.621 | 0.621 | 27 |
| ALB | <0.0001 | 0.0002 | 0.657 | -0.606 | 0.606 | 28 |

**Table S4. DEPs identified from the comparison between HLH and healthy controls**

| Gene Names | P value | Adjusted P value | Fold change | log2fold | abs_lg2_fd | Fold change rank |
| --- | --- | --- | --- | --- | --- | --- |
| ERN1 | 0.0015 | 0.0095 | 23.913 | 4.580 | 4.580 | 1 |
| S100A8 | 0.0106 | 0.0489 | 14.778 | 3.885 | 3.885 | 2 |
| H2AX | 0.0033 | 0.0188 | 12.951 | 3.695 | 3.695 | 3 |
| ANTXR1 | 0.0101 | 0.0472 | 0.082 | -3.602 | 3.602 | 4 |
| SAA1 | 0.0046 | 0.0257 | 7.732 | 2.951 | 2.951 | 5 |
| CRP | 0.0006 | 0.0047 | 7.480 | 2.903 | 2.903 | 6 |
| FCGR3A | <0.0001 | <0.0001 | 6.867 | 2.780 | 2.780 | 7 |
| SAA2 | 0.0097 | 0.0459 | 6.741 | 2.753 | 2.753 | 8 |
| FCGR2A | <0.0001 | <0.0001 | 6.271 | 2.649 | 2.649 | 9 |
| H4C1 | 0.0095 | 0.0452 | 6.139 | 2.618 | 2.618 | 10 |
| UBE2L6 | 0.0014 | 0.0091 | 5.990 | 2.583 | 2.583 | 11 |
| ACTB | <0.0001 | <0.0001 | 5.443 | 2.444 | 2.444 | 12 |
| H2BC11 | 0.0088 | 0.0422 | 5.116 | 2.355 | 2.355 | 13 |
| LCP1 | <0.0001 | <0.0001 | 4.540 | 2.183 | 2.183 | 14 |
| PIGR | <0.0001 | 0.0001 | 4.478 | 2.163 | 2.163 | 15 |
| B2M | <0.0001 | 0.0002 | 4.256 | 2.090 | 2.090 | 16 |
| DEFA3 | <0.0001 | <0.0001 | 4.098 | 2.035 | 2.035 | 17 |
| ACTG1 | 0.0006 | 0.0048 | 4.087 | 2.031 | 2.031 | 18 |
| ACTA2 | 0.0010 | 0.0067 | 3.355 | 1.746 | 1.746 | 19 |
| IGFALS | <0.0001 | <0.0001 | 0.300 | -1.738 | 1.738 | 20 |
| APOC1 | <0.0001 | <0.0001 | 0.308 | -1.701 | 1.701 | 21 |
| SELENOP | <0.0001 | <0.0001 | 0.315 | -1.665 | 1.665 | 22 |
| N4BP2 | 0.0009 | 0.0066 | 0.336 | -1.574 | 1.574 | 23 |
| H2BC12 | 0.0096 | 0.0458 | 2.972 | 1.572 | 1.572 | 24 |
| ICAM1 | <0.0001 | 0.0001 | 2.790 | 1.480 | 1.480 | 25 |
| LUM | <0.0001 | <0.0001 | 0.363 | -1.461 | 1.461 | 26 |
| CORO1A | 0.0077 | 0.0380 | 2.702 | 1.434 | 1.434 | 27 |
| APOA1 | <0.0001 | <0.0001 | 0.372 | -1.428 | 1.428 | 28 |
| ROR1 | <0.0001 | <0.0001 | 0.373 | -1.423 | 1.423 | 29 |
| CD163 | 0.0006 | 0.0044 | 2.671 | 1.417 | 1.417 | 30 |
| LBP | <0.0001 | <0.0001 | 2.529 | 1.339 | 1.339 | 31 |
| IGFBP3 | <0.0001 | 0.0001 | 0.396 | -1.335 | 1.335 | 32 |
| IGHV4OR15-8 | 0.0003 | 0.0023 | 2.503 | 1.324 | 1.324 | 33 |
| SERPINA5 | <0.0001 | <0.0001 | 0.411 | -1.282 | 1.282 | 34 |
| VCAM1 | <0.0001 | <0.0001 | 2.423 | 1.277 | 1.277 | 35 |
| HSP90AB1 | 0.0009 | 0.0066 | 2.335 | 1.224 | 1.224 | 36 |
| HLA-A | 0.0007 | 0.0050 | 2.321 | 1.215 | 1.215 | 37 |
| CD14 | <0.0001 | 0.0005 | 2.295 | 1.199 | 1.199 | 38 |
| PRDX1 | 0.0004 | 0.0030 | 2.284 | 1.192 | 1.192 | 39 |
| A2M | <0.0001 | <0.0001 | 0.441 | -1.181 | 1.181 | 40 |
| IL18BP | <0.0001 | <0.0001 | 2.265 | 1.180 | 1.180 | 41 |
| ECM1 | <0.0001 | <0.0001 | 0.444 | -1.170 | 1.170 | 42 |
| MSN | 0.0004 | 0.0031 | 2.238 | 1.162 | 1.162 | 43 |
| LGALS3BP | <0.0001 | <0.0001 | 2.226 | 1.155 | 1.155 | 44 |
| SERPINA4 | <0.0001 | <0.0001 | 0.458 | -1.126 | 1.126 | 45 |
| HRG | <0.0001 | <0.0001 | 0.465 | -1.105 | 1.105 | 46 |
| AHSG | <0.0001 | <0.0001 | 0.466 | -1.101 | 1.101 | 47 |
| ALB | <0.0001 | <0.0001 | 0.466 | -1.100 | 1.100 | 48 |
| HSP90AA1 | 0.0001 | 0.0006 | 2.142 | 1.099 | 1.099 | 49 |
| GSN | <0.0001 | <0.0001 | 0.474 | -1.077 | 1.077 | 50 |
| VCAN | 0.0011 | 0.0073 | 2.106 | 1.075 | 1.075 | 51 |
| YWHAB | 0.0001 | 0.0010 | 2.082 | 1.058 | 1.058 | 52 |
| ITIH2 | <0.0001 | <0.0001 | 0.483 | -1.051 | 1.051 | 53 |
| ORM1 | <0.0001 | 0.0002 | 2.067 | 1.048 | 1.048 | 54 |
| APOM | <0.0001 | <0.0001 | 0.487 | -1.039 | 1.039 | 55 |
| TF | <0.0001 | <0.0001 | 0.491 | -1.027 | 1.027 | 56 |
| APOA4 | <0.0001 | <0.0001 | 0.495 | -1.015 | 1.015 | 57 |
| CPB2 | <0.0001 | <0.0001 | 0.498 | -1.005 | 1.005 | 58 |
| CBLN4 | 0.0026 | 0.0155 | 0.499 | -1.004 | 1.004 | 59 |
| PON1 | <0.0001 | <0.0001 | 0.500 | -1.000 | 1.000 | 60 |
| CLEC3B | <0.0001 | <0.0001 | 0.504 | -0.990 | 0.990 | 61 |
| GPLD1 | <0.0001 | <0.0001 | 0.508 | -0.978 | 0.978 | 62 |
| STXBP5 | 0.0064 | 0.0334 | 0.508 | -0.977 | 0.977 | 63 |
| BCHE | <0.0001 | <0.0001 | 0.508 | -0.977 | 0.977 | 64 |
| ENO1 | 0.0003 | 0.0023 | 1.965 | 0.974 | 0.974 | 65 |
| ADA2 | <0.0001 | 0.0002 | 1.936 | 0.953 | 0.953 | 66 |
| LRG1 | <0.0001 | <0.0001 | 1.928 | 0.947 | 0.947 | 67 |
| CD5L | 0.0001 | 0.0008 | 0.520 | -0.944 | 0.944 | 68 |
| HGFAC | <0.0001 | <0.0001 | 0.521 | -0.940 | 0.940 | 69 |
| TYMP | <0.0001 | 0.0001 | 1.914 | 0.936 | 0.936 | 70 |
| CAT | <0.0001 | <0.0001 | 1.903 | 0.928 | 0.928 | 71 |
| APOH | <0.0001 | <0.0001 | 0.527 | -0.924 | 0.924 | 72 |
| RBP4 | <0.0001 | <0.0001 | 0.528 | -0.922 | 0.922 | 73 |
| CFHR4 | 0.0029 | 0.0167 | 0.533 | -0.907 | 0.907 | 74 |
| SFTPB | 0.0101 | 0.0472 | 1.874 | 0.906 | 0.906 | 75 |
| CETP | <0.0001 | <0.0001 | 0.542 | -0.885 | 0.885 | 76 |
| CD99 | 0.0069 | 0.0348 | 0.542 | -0.883 | 0.883 | 77 |
| PSMA3 | 0.0076 | 0.0377 | 1.840 | 0.880 | 0.880 | 78 |
| TTR | <0.0001 | <0.0001 | 0.545 | -0.877 | 0.877 | 79 |
| KRT2 | 0.0001 | 0.0006 | 0.551 | -0.861 | 0.861 | 80 |
| OSMR | 0.0030 | 0.0175 | 1.807 | 0.854 | 0.854 | 81 |
| YWHAG | 0.0002 | 0.0020 | 1.803 | 0.851 | 0.851 | 82 |
| YWHAE | <0.0001 | <0.0001 | 1.801 | 0.849 | 0.849 | 83 |
| SAA4 | <0.0001 | <0.0001 | 0.556 | -0.848 | 0.848 | 84 |
| FGB | <0.0001 | <0.0001 | 0.556 | -0.846 | 0.846 | 85 |
| SERPIND1 | <0.0001 | <0.0001 | 0.562 | -0.832 | 0.832 | 86 |
| LAP3 | 0.0001 | 0.0006 | 1.769 | 0.823 | 0.823 | 87 |
| WARS1 | <0.0001 | 0.0001 | 1.753 | 0.810 | 0.810 | 88 |
| AFM | <0.0001 | <0.0001 | 0.570 | -0.810 | 0.810 | 89 |
| APOA2 | <0.0001 | <0.0001 | 0.573 | -0.803 | 0.803 | 90 |
| PSMB4 | 0.0078 | 0.0385 | 1.733 | 0.793 | 0.793 | 91 |
| KRT1 | 0.0001 | 0.0005 | 0.579 | -0.789 | 0.789 | 92 |
| PFN1 | 0.0004 | 0.0030 | 1.720 | 0.783 | 0.783 | 93 |
| RPLP1 | 0.0047 | 0.0261 | 1.719 | 0.782 | 0.782 | 94 |
| F2 | <0.0001 | <0.0001 | 0.583 | -0.779 | 0.779 | 95 |
| FGG | <0.0001 | <0.0001 | 0.584 | -0.775 | 0.775 | 96 |
| GP1BA | 0.0067 | 0.0344 | 1.704 | 0.769 | 0.769 | 97 |
| KRT9 | 0.0001 | 0.0013 | 0.591 | -0.759 | 0.759 | 98 |
| CTSD | 0.0020 | 0.0122 | 1.685 | 0.753 | 0.753 | 99 |
| FGA | <0.0001 | <0.0001 | 0.594 | -0.751 | 0.751 | 100 |
| EEF2 | 0.0095 | 0.0452 | 1.677 | 0.746 | 0.746 | 101 |
| SH3BGRL3 | <0.0001 | 0.0001 | 1.675 | 0.745 | 0.745 | 102 |
| GSTO1 | <0.0001 | <0.0001 | 1.675 | 0.744 | 0.744 | 103 |
| ATRN | <0.0001 | <0.0001 | 0.599 | -0.740 | 0.740 | 104 |
| KLKB1 | <0.0001 | <0.0001 | 0.600 | -0.736 | 0.736 | 105 |
| CSF1R | 0.0042 | 0.0236 | 1.656 | 0.728 | 0.728 | 106 |
| PSMA5 | 0.0003 | 0.0022 | 1.652 | 0.724 | 0.724 | 107 |
| KRT10 | <0.0001 | 0.0004 | 0.608 | -0.717 | 0.717 | 108 |
| OAF | <0.0001 | <0.0001 | 1.639 | 0.713 | 0.713 | 109 |
| TKT | 0.0002 | 0.0018 | 1.625 | 0.701 | 0.701 | 110 |
| SHBG | 0.0010 | 0.0067 | 0.617 | -0.696 | 0.696 | 111 |
| FABP5 | 0.0019 | 0.0114 | 1.614 | 0.691 | 0.691 | 112 |
| GSS | 0.0061 | 0.0317 | 1.612 | 0.689 | 0.689 | 113 |
| SPINK1 | 0.0084 | 0.0414 | 1.609 | 0.687 | 0.687 | 114 |
| PGLYRP2 | <0.0001 | <0.0001 | 0.622 | -0.686 | 0.686 | 115 |
| UBB | 0.0012 | 0.0080 | 1.603 | 0.681 | 0.681 | 116 |
| ITIH1 | <0.0001 | <0.0001 | 0.627 | -0.673 | 0.673 | 117 |
| YWHAZ | <0.0001 | <0.0001 | 1.592 | 0.671 | 0.671 | 118 |
| GAPDH | 0.0001 | 0.0007 | 1.589 | 0.668 | 0.668 | 119 |
| IGFBP2 | 0.0003 | 0.0024 | 1.588 | 0.667 | 0.667 | 120 |
| ICAM3 | 0.0053 | 0.0286 | 1.580 | 0.660 | 0.660 | 121 |
| LDHA | <0.0001 | <0.0001 | 1.573 | 0.653 | 0.653 | 122 |
| IGF2 | <0.0001 | <0.0001 | 0.637 | -0.650 | 0.650 | 123 |
| ALDOA | <0.0001 | <0.0001 | 1.568 | 0.649 | 0.649 | 124 |
| TALDO1 | 0.0005 | 0.0040 | 1.563 | 0.644 | 0.644 | 125 |
| CFHR1 | <0.0001 | <0.0001 | 0.641 | -0.641 | 0.641 | 126 |
| HSPA8 | 0.0001 | 0.0011 | 1.554 | 0.636 | 0.636 | 127 |
| SOD2 | 0.0020 | 0.0120 | 1.548 | 0.630 | 0.630 | 128 |
| LYZ | 0.0007 | 0.0053 | 1.547 | 0.630 | 0.630 | 129 |
| VWF | <0.0001 | <0.0001 | 1.534 | 0.617 | 0.617 | 130 |
| PI16 | <0.0001 | <0.0001 | 0.652 | -0.616 | 0.616 | 131 |
| SERPINC1 | <0.0001 | <0.0001 | 0.653 | -0.614 | 0.614 | 132 |
| NID1 | <0.0001 | 0.0001 | 1.528 | 0.611 | 0.611 | 133 |
| GC | <0.0001 | <0.0001 | 0.655 | -0.611 | 0.611 | 134 |
| C3 | <0.0001 | <0.0001 | 0.655 | -0.610 | 0.610 | 135 |
| PROS1 | <0.0001 | <0.0001 | 0.655 | -0.609 | 0.609 | 136 |
| SERPINA6 | <0.0001 | <0.0001 | 0.658 | -0.603 | 0.603 | 137 |
| C8G | <0.0001 | <0.0001 | 0.662 | -0.595 | 0.595 | 138 |
| FN1 | <0.0001 | 0.0003 | 0.662 | -0.595 | 0.595 | 139 |
| LCAT | <0.0001 | <0.0001 | 0.669 | -0.580 | 0.580 | 140 |

**Table S5. DEPs identified from the comparison between HLH and patients from the PICU without infection**

| Gene Names | P value | Adjusted P value | Fold change | log2fold | abs_lg2_fd | Fold change rank |
| --- | --- | --- | --- | --- | --- | --- |
| ERN1 | 0.0017 | 0.0137 | 19.070 | 4.253 | 4.253 | 1 |
| PF4V1 | <0.0001 | <0.0001 | 0.177 | -2.498 | 2.498 | 2 |
| FCGR3A | <0.0001 | <0.0001 | 4.833 | 2.273 | 2.273 | 3 |
| ACTG1 | 0.0004 | 0.0041 | 4.817 | 2.268 | 2.268 | 4 |
| UBE2L6 | 0.0024 | 0.0185 | 4.716 | 2.238 | 2.238 | 5 |
| PPBP | <0.0001 | <0.0001 | 0.219 | -2.191 | 2.191 | 6 |
| ACTB | <0.0001 | 0.0001 | 4.108 | 2.038 | 2.038 | 7 |
| LCP1 | <0.0001 | <0.0001 | 3.767 | 1.914 | 1.914 | 8 |
| PIGR | <0.0001 | 0.0007 | 3.489 | 1.803 | 1.803 | 9 |
| FCGR2A | 0.0001 | 0.0013 | 3.380 | 1.757 | 1.757 | 10 |
| ACTA2 | 0.0014 | 0.0116 | 3.131 | 1.646 | 1.646 | 11 |
| SELENOP | <0.0001 | <0.0001 | 0.337 | -1.568 | 1.568 | 12 |
| APOC1 | <0.0001 | <0.0001 | 0.343 | -1.544 | 1.544 | 13 |
| ICAM1 | <0.0001 | 0.0002 | 2.654 | 1.408 | 1.408 | 14 |
| B2M | 0.0003 | 0.0040 | 2.653 | 1.408 | 1.408 | 15 |
| CD163 | 0.0008 | 0.0079 | 2.553 | 1.352 | 1.352 | 16 |
|  | 0.0045 | 0.0316 | 0.407 | -1.297 | 1.297 | 17 |
| APOA1 | <0.0001 | <0.0001 | 0.411 | -1.283 | 1.283 | 18 |
| LUM | <0.0001 | <0.0001 | 0.419 | -1.255 | 1.255 | 19 |
| PI16 | 0.0017 | 0.0140 | 0.420 | -1.252 | 1.252 | 20 |
| IGHV1-69D | 0.0035 | 0.0258 | 0.440 | -1.185 | 1.185 | 21 |
| AHSG | <0.0001 | <0.0001 | 0.449 | -1.155 | 1.155 | 22 |
| HSP90AB1 | 0.0014 | 0.0118 | 2.225 | 1.154 | 1.154 | 23 |
| IGHV3-43 | 0.0005 | 0.0051 | 0.455 | -1.136 | 1.136 | 24 |
| VCAM1 | <0.0001 | <0.0001 | 2.155 | 1.107 | 1.107 | 25 |
| DEFA3 | 0.0007 | 0.0068 | 2.128 | 1.089 | 1.089 | 26 |
| SERPINA5 | <0.0001 | <0.0001 | 0.471 | -1.085 | 1.085 | 27 |
| CD14 | 0.0002 | 0.0030 | 2.119 | 1.084 | 1.084 | 28 |
| CFHR4 | 0.0007 | 0.0069 | 0.479 | -1.062 | 1.062 | 29 |
| PRDX1 | 0.0010 | 0.0096 | 2.066 | 1.047 | 1.047 | 30 |
| IGFALS | <0.0001 | <0.0001 | 0.484 | -1.046 | 1.046 | 31 |
| HLA-A | 0.0022 | 0.0174 | 2.045 | 1.032 | 1.032 | 32 |
| ROR1 | 0.0002 | 0.0024 | 0.491 | -1.026 | 1.026 | 33 |
| MSN | 0.0011 | 0.0099 | 2.032 | 1.023 | 1.023 | 34 |
| CRISP3 | 0.0006 | 0.0061 | 0.493 | -1.021 | 1.021 | 35 |
| A2M | <0.0001 | <0.0001 | 0.494 | -1.016 | 1.016 | 36 |
| ECM1 | <0.0001 | <0.0001 | 0.495 | -1.015 | 1.015 | 37 |
| HSP90AA1 | 0.0001 | 0.0018 | 2.015 | 1.010 | 1.010 | 38 |
| LBP | <0.0001 | 0.0007 | 1.976 | 0.982 | 0.982 | 39 |
| ALB | <0.0001 | <0.0001 | 0.508 | -0.978 | 0.978 | 40 |
| IGLV6-57 | 0.0038 | 0.0278 | 0.509 | -0.975 | 0.975 | 41 |
| APOM | <0.0001 | <0.0001 | 0.515 | -0.958 | 0.958 | 42 |
| APOH | <0.0001 | <0.0001 | 0.516 | -0.955 | 0.955 | 43 |
| HRG | <0.0001 | <0.0001 | 0.522 | -0.937 | 0.937 | 44 |
| ADIPOQ | 0.0055 | 0.0372 | 0.525 | -0.929 | 0.929 | 45 |
| LGALS3BP | <0.0001 | 0.0005 | 1.878 | 0.909 | 0.909 | 46 |
| TYMP | <0.0001 | 0.0001 | 1.872 | 0.905 | 0.905 | 47 |
| BCHE | <0.0001 | <0.0001 | 0.535 | -0.903 | 0.903 | 48 |
| THBS1 | <0.0001 | 0.0001 | 0.537 | -0.897 | 0.897 | 49 |
| IGKV2D-24 | 0.0012 | 0.0107 | 0.539 | -0.892 | 0.892 | 50 |
| YWHAB | 0.0005 | 0.0058 | 1.844 | 0.883 | 0.883 | 51 |
| CD5L | 0.0005 | 0.0049 | 0.547 | -0.870 | 0.870 | 52 |
| TF | <0.0001 | <0.0001 | 0.548 | -0.868 | 0.868 | 53 |
| ITIH2 | <0.0001 | <0.0001 | 0.549 | -0.865 | 0.865 | 54 |
| HGFAC | <0.0001 | <0.0001 | 0.550 | -0.862 | 0.862 | 55 |
| SERPINA4 | <0.0001 | <0.0001 | 0.555 | -0.850 | 0.850 | 56 |
| IL18BP | <0.0001 | 0.0002 | 1.798 | 0.847 | 0.847 | 57 |
| ADA2 | 0.0001 | 0.0014 | 1.776 | 0.829 | 0.829 | 58 |
| GSN | <0.0001 | <0.0001 | 0.567 | -0.818 | 0.818 | 59 |
| CLEC3B | <0.0001 | <0.0001 | 0.568 | -0.816 | 0.816 | 60 |
| TTR | <0.0001 | <0.0001 | 0.568 | -0.816 | 0.816 | 61 |
| LAP3 | 0.0001 | 0.0011 | 1.758 | 0.814 | 0.814 | 62 |
| WARS1 | <0.0001 | 0.0001 | 1.755 | 0.811 | 0.811 | 63 |
| IGFBP3 | <0.0001 | <0.0001 | 0.571 | -0.809 | 0.809 | 64 |
| IGF2 | <0.0001 | <0.0001 | 0.573 | -0.803 | 0.803 | 65 |
| SAA4 | <0.0001 | <0.0001 | 0.577 | -0.793 | 0.793 | 66 |
| YWHAG | 0.0005 | 0.0055 | 1.720 | 0.782 | 0.782 | 67 |
| LRG1 | <0.0001 | 0.0002 | 1.719 | 0.781 | 0.781 | 68 |
| ENO1 | 0.0027 | 0.0207 | 1.683 | 0.751 | 0.751 | 69 |
| GPLD1 | <0.0001 | <0.0001 | 0.595 | -0.749 | 0.749 | 70 |
| PON1 | <0.0001 | <0.0001 | 0.601 | -0.735 | 0.735 | 71 |
| APOA2 | <0.0001 | <0.0001 | 0.609 | -0.715 | 0.715 | 72 |
| ATRN | <0.0001 | <0.0001 | 0.618 | -0.694 | 0.694 | 73 |
| RBP4 | 0.0001 | 0.0019 | 0.620 | -0.690 | 0.690 | 74 |
| CPB2 | <0.0001 | <0.0001 | 0.621 | -0.687 | 0.687 | 75 |
| FGB | 0.0001 | 0.0014 | 0.624 | -0.680 | 0.680 | 76 |
| APOA4 | 0.0003 | 0.0035 | 0.625 | -0.677 | 0.677 | 77 |
| YWHAE | 0.0001 | 0.0011 | 1.585 | 0.664 | 0.664 | 78 |
| FGG | <0.0001 | 0.0004 | 0.634 | -0.658 | 0.658 | 79 |
| KLKB1 | <0.0001 | <0.0001 | 0.645 | -0.633 | 0.633 | 80 |
| PGLYRP2 | <0.0001 | <0.0001 | 0.649 | -0.624 | 0.624 | 81 |
| GSTO1 | <0.0001 | <0.0001 | 1.527 | 0.611 | 0.611 | 82 |
| APMAP | 0.0002 | 0.0029 | 0.655 | -0.610 | 0.610 | 83 |
| CETP | <0.0001 | <0.0001 | 0.662 | -0.596 | 0.596 | 84 |
| SHBG | <0.0001 | 0.0006 | 0.663 | -0.593 | 0.593 | 85 |
| AFM | <0.0001 | <0.0001 | 0.663 | -0.592 | 0.592 | 86 |

**Table S6. DEPs identified from the comparison between EBV-HLH and EBV non-HLH**

| Gene Names | P value | Adjusted P value | Fold change | log2fold | abs_lg2_fd | Fold change rank |
| --- | --- | --- | --- | --- | --- | --- |
| HP | 0.0023 | 0.0469 | 0.301 | -1.733 | 1.733 | 1 |
| HPR | 0.0001 | 0.0091 | 0.350 | -1.515 | 1.515 | 2 |
| LCP1 | 0.0002 | 0.0106 | 2.814 | 1.492 | 1.492 | 3 |
| CD5L | 0.0013 | 0.0355 | 0.402 | -1.314 | 1.314 | 4 |
| IGHV3-43 | 0.0022 | 0.0461 | 0.406 | -1.301 | 1.301 | 5 |
| IGLV6-57 | <0.0001 | 0.0014 | 0.423 | -1.240 | 1.240 | 6 |
| ECM1 | <0.0001 | 0.0061 | 0.445 | -1.167 | 1.167 | 7 |
| SELENOP | 0.0001 | 0.0091 | 0.467 | -1.100 | 1.100 | 8 |
| IGHV4-28 | 0.0018 | 0.0432 | 0.496 | -1.011 | 1.011 | 9 |
| IGHV3-73 | 0.0001 | 0.0095 | 0.515 | -0.957 | 0.957 | 10 |
| APOA1 | <0.0001 | 0.0020 | 0.539 | -0.892 | 0.892 | 11 |
| SAA4 | 0.0001 | 0.0076 | 0.547 | -0.870 | 0.870 | 12 |
| APOC1 | 0.0005 | 0.0188 | 0.552 | -0.856 | 0.856 | 13 |
| A2M | <0.0001 | 0.0007 | 0.566 | -0.822 | 0.822 | 14 |
| AHSG | 0.0001 | 0.0091 | 0.566 | -0.820 | 0.820 | 15 |
| ALB | <0.0001 | 0.0009 | 0.590 | -0.760 | 0.760 | 16 |
| FGA | 0.0005 | 0.0190 | 0.591 | -0.760 | 0.760 | 17 |
| FGG | 0.0002 | 0.0111 | 0.594 | -0.750 | 0.750 | 18 |
| VCAM1 | 0.0006 | 0.0205 | 1.623 | 0.699 | 0.699 | 19 |
| FGB | 0.0025 | 0.0485 | 0.623 | -0.682 | 0.682 | 20 |
| CETP | 0.0003 | 0.0143 | 0.634 | -0.657 | 0.657 | 21 |
| TF | 0.0001 | 0.0091 | 0.637 | -0.651 | 0.651 | 22 |
| SPINK1 | 0.0015 | 0.0383 | 0.638 | -0.649 | 0.649 | 23 |
| TTR | 0.0002 | 0.0106 | 0.640 | -0.643 | 0.643 | 24 |
| SERPINA5 | 0.0005 | 0.0189 | 0.652 | -0.618 | 0.618 | 25 |
| FN1 | 0.0022 | 0.0461 | 0.660 | -0.600 | 0.600 | 26 |
| APOH | 0.0002 | 0.0102 | 0.669 | -0.581 | 0.581 | 27 |
