# Supplementary material for: Proteomic Analysis of Pediatric Hemophagocytic Lymphohistiocytosis: a Comparative Study with Healthy Controls, Sepsis, Critical Ill, and Active Epstein-Barr virus Infection to Identify Altered Pathways and Candidate Biomarkers
Source: J Clin Immunol. 2023 Aug 31;43(8):1997–2010. doi: 10.1007/s10875-023-01573-w (PMC10661879; doi:10.1007/s10875-023-01573-w)
Supplement: Supplementary file 3 — Supplementary information 3 Figure S1. Quality control results of indexed retention time/retention time, Figure S2. Correlations between proteomic quantification of FGA, FGG, and clinically tested fibrinogen levels (DOCX 261 kb) [file 10875_2023_1573_MOESM3_ESM.docx]

**Supplementary File 3**

**Supplementary Figures**


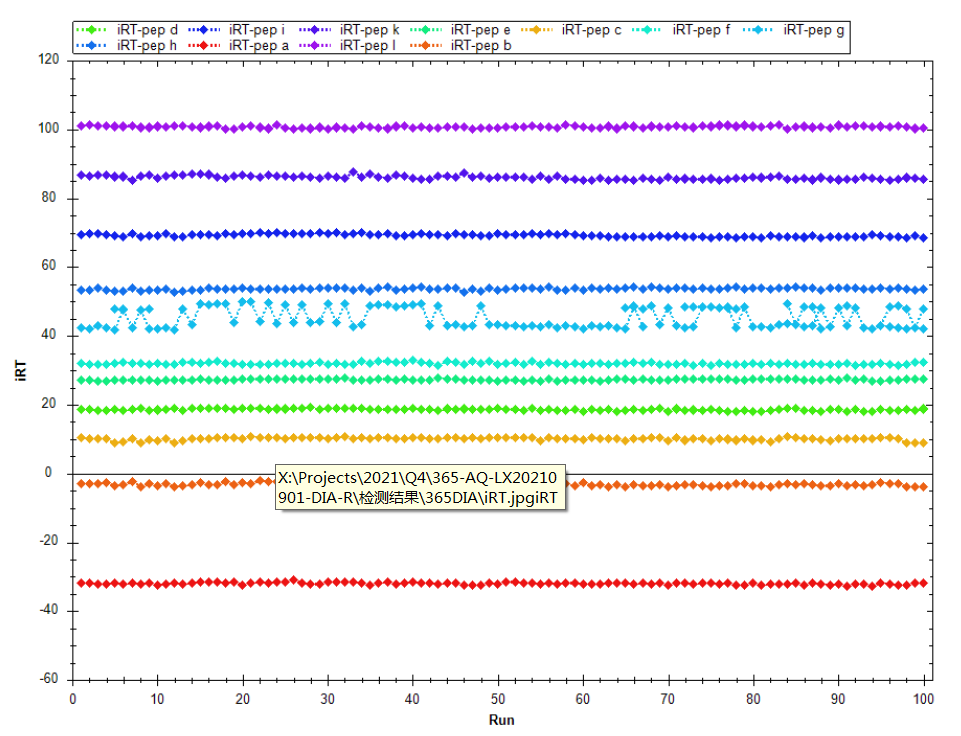


**Figure S1. Quality control results of indexed retention time/retention time**


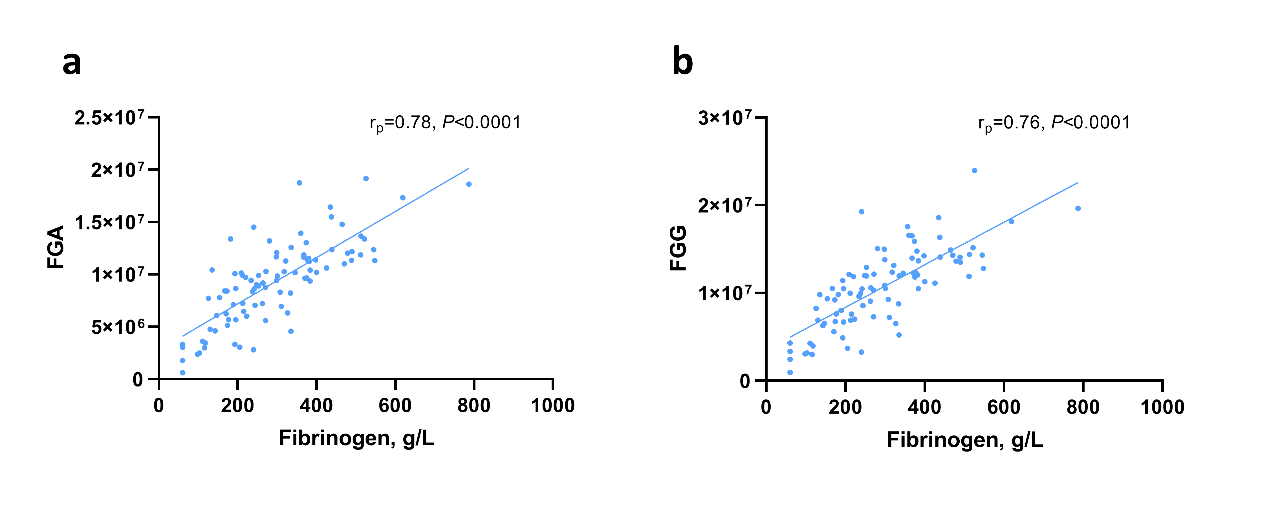


**Figure S2. Correlations between proteomic quantification of FGA, FGG, and clinically tested fibrinogen levels.** FGA, fibrinogen alpha chain; FGG, fibrinogen gamma chain.
